# A province-by-province cost-effectiveness analysis and budget impact analysis of one-time birth cohort screening of hepatitis C virus (HCV) infection in Canada

**DOI:** 10.1038/s41598-023-39521-8

**Published:** 2023-08-18

**Authors:** William W. L. Wong, Alex Haines, Josephine Wong, Abdullah Hamadeh, Murray D. Krahn

**Affiliations:** 1 School of Pharmacy, Faculty of Science, University of Waterloo, PHR4011, 10A Victoria Street S, Kitchener, ON N2G1C5 Canada; 2 Toronto Health Economics and Technology Assessment Collaborative (THETA), University Health Network, Toronto, ON Canada; 3 ICES, Toronto, ON Canada

**Keywords:** Hepatitis, Health care economics

## Abstract

Managing chronic hepatitis C is challenging, as the majority of those infected are asymptomatic. Therefore, to ensure treatments are administered before the onset of severe complications, screening is important. In Canada, uncertainty regarding the cost-effectiveness and budget impact of screening has led to conflicting recommendations. The objective of this study is to estimate the cost-effectiveness and budget-impact of one-time HCV screening. A state-transition model was developed to evaluate the cost-effectiveness and budget-impact between a risk-based screening strategy (current-practice) and a one-time screening strategy on three different birth-cohorts. Cost and prevalence data were obtained from administrative data. Progression and utility data were based on recent systematic reviews. We used a provincial payer-perspective, life-time time-horizon and a 1.5% discount rate for the cost-effectiveness analysis, and used a 10-year time-horizon and no discounting for the budget-impact analysis. One-time screening strategy would cost more and provide more health benefits than the risk-based screening for all birth cohorts. For those born after 1964, the incremental-cost-effectiveness-ratio (ICER) per quality-adjusted-life-year (QALY) of screening versus current-practice varied from $27,422/QALY to $42,191/QALY across different provinces. One-time screening of the cohort would cost an additional $2 million to $236 million across different provinces. For those born 1945–1964, the ICER of screening versus current-practice varied from $35,217/QALY to $48,197/QALY across different provinces. For the cohort born before 1945, the ICER of screening versus current-practice was not cost-effective at a willingness-to-pay threshold of $50,000/QALY across all provinces. Our cost-effectiveness analysis suggests that a one-time HCV screening program for those born after 1945 is cost-effective. Considering the budget impact relative to other funded recommended health services and technologies, HCV screening could be considered affordable.

## Introduction

Managing Chronic Hepatitis C (CHC) is challenging because majority of those infected are asymptomatic^1^. Screening can potentially identify people who are asymptomatic and allow medical treatment to be offered when necessary^2^. In 2016, Canada agreed to meet the targets put forward by the World Health Organization of reducing new cases of hepatitis C (HCV) by 90%, hepatitis-related deaths by 65%, and treatment of 80% of eligible cases by 2030^3–5^. Since then, most of the provinces have approved full coverage of the Direct Acting Antiviral (DAA) treatment for all chronic hepatitis C (CHC) patients^6,7^. However, there exist conflicting recommendations in terms of HCV screening. One proposed initiative is to screen all individuals born between 1945 and 1964, also known as ‘baby-boomers’, as evidence shows that prevalence is the highest in this age cohort^8,9^. Screening this cohort has been advocated by the Canadian Association for the Study of the Liver^10,11^ and the Centers for Disease Control and Prevention (CDC)^12^ in the United States. CDC now recommends one-time HCV screening of all adults and all pregnant women in addition to people with risk factors^13^. However, the Canadian Taskforce for Preventive Healthcare (CTFPHC) issued recommendations against HCV screening in adults who are not at elevated risk citing potential harm of stigma and issues with evidence, equity and budget impact^2^.

In 2014, we built a policy model in collaboration with Canadian Agency for Drugs and Technologies in Health (CADTH) and Public Health Agency of Canada to assess the cost-effectiveness of the first generation of DAA and the cost-effectiveness of one-time hepatitis C screening in Canada respectively^14,15^. Since then, the model was used by CADTH and CTFPHC to determine the currently in-place recommendations related to hepatitis C treatment and screening^16^. In terms of one-time HCV screening, although the model indicated a reasonable chance of being cost-effective, particularly for the “baby-bloomer” population, sensitivity analyses indicated considerable amount of uncertainty associated with such conclusion especially when the prevalence, the undiagnosed proportion, the cost, and the utilities of the disease were not known^15–17^.

Newly available evidence have published over the past five years that may have influenced the cost-effectiveness of one-time HCV screening. This included a back-calculation mathematical model to project recent prevalence and undiagnosed proportion using health-administrative data^18^; a retrospective analysis of health-administrative data from a population cohort with CHC to generate health-states specific costs for modeling^19^; two recent systematic review updates on CHC progression data and CHC utility data^20,21^; and qualitative evidence that summarized the current and changing landscape of HCV care^22^. The availability of these resources has allowed us to develop a more comprehensive HCV screening model that integrated the up-to-date evidence to estimate the cost-effectiveness and the budget-impact of a one-time HCV screening program for each Canadian province.

## Materials and methods

We developed a state-transition model of HCV to assess the cost-effectiveness and budget impact of three birth-cohort HCV screening strategies for each of the ten Canadian provinces. An overview of our method will be presented in the following subsections. A detailed methodology section can be found in Supplementary Information S1.

### Study cohorts

Three birth cohorts were considered in the analysis; which, when combined, covered all individuals over 18 years of age living in Canada. They were: (1) *people born after 1964 and over 18 years of age*; (2) *people born between 1945 and 1964*; and (3) *people born before 1945*. The mean age of the cohort was 39, 58 and 78, respectively^19,23^.

### Screening strategies

HCV screening involves a HCV antibody test, which if positive, will be followed up with a HCV RNA test to confirm the infection. We considered two screening strategies for the base-case analyses:

- Status quo: Screening high risk individuals only, based on current recommendation from the Public Health Agency of Canada^24,25^. Screening was offered to anyone with a risk factor (such as injection drug user; received tattooing or piercing; blood exposure during sex or from non-sterile equipment; received blood product or organ transplant prior to 1992; born, visited or resided in hepatitis C endemic regions; born to mother with hepatitis C; or shared personal items with hepatitis C patients) or a clinical indication (such as HIV or hepatitis B diagnosis; symptoms of liver disease; occupational exposure).
- A “case finding” one time *screening*: Individuals born within the eligible years were offered one-time HCV screening by their primary care physician at a visit scheduled for another purpose. We considered this intervention for the three study cohorts.

### Decision model

**Figure 1:**
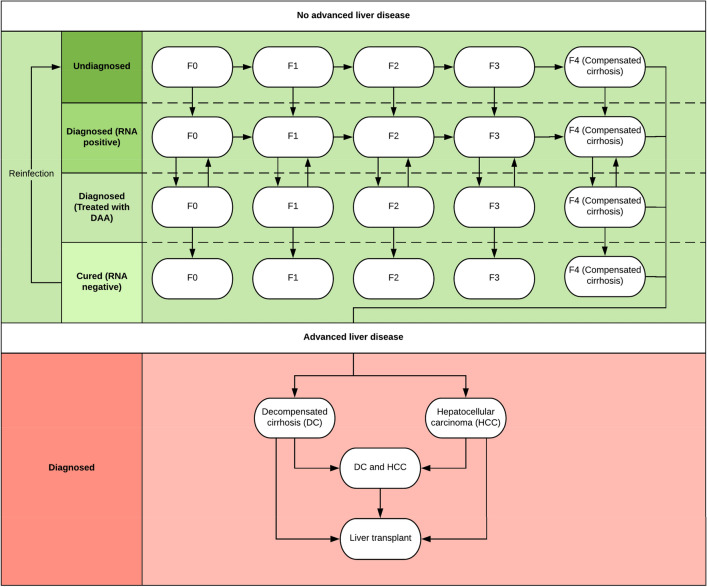
State transition model of the natural history of hepatitis C infection.

We constructed a state-based transition model^26^ with health states that reflected the natural history of CHC from acute infection to end stage liver disease as illustrated in Fig. 1. The model simulated the transition through health states over time for a given cohort using monthly time intervals. TreeAge Pro 2021^21^ was used to construct the model**.** In the model, the cohort being simulated was all individuals who met the criteria for HCV screening. Individuals could be diagnosed with HCV at any point. The probability of HCV diagnosis was contingent on the screening policies in place. Once diagnosed with HCV, the individuals entered a new state with the same fibrosis stage and with a chance of receiving HCV treatment^27^. Patients were assumed to be cured if they achieved a sustained virologic response at 12 weeks post-treatment^28,29^. Once an individual became cured, there was a probability that they might become re-infected if they continued to partake in high risk activities such as illicit drug use^30^.

### Model parameters

Each province has its own parameters on prevalence, undiagnosed proportion, fibrosis distribution and HCV related cost for each health states. Prevalence and undiagnosed proportion estimates were obtained from a back-calculation model, utilizing health administrative data from British Columbia and Ontario^8,18^. The back-calculation model employed a mathematical algorithm was used to retrospectively estimate historical CHC prevalence through a calibration process. HCV related cost data were obtained from administrative data in Ontario, and we used data from the Canadian Institute for Health Information to calculate the cost difference across provinces^19^. Natural history progression data and utility data were based on two systematic reviews published in 2019^20,21^. Key parameters were listed in Table 1 with details presented in Supplementary Information S1.

**Table 1:** Model parameters.

Model parameter	Value (range)	Source
Prevalence of CHC		Hamadeh et al.^8,31,32^
People born before 1945		
BC	0.75% (0.59–0.99%)	
AB/SK/MB	1.60% (1.11–2.43%)	
ON	0.75% (0.60–1.06%)	
QC	1.03% (0.64–1.61%)	
NB/NS/PE/NL	2.09% (1.18–3.48%)	
People born between 1945 and 1964		
BC	2.31% (1.71–3.68%)	
AB/SK/MB	2.24% (1.73–3.04%)	
ON	1.93% (1.69–2.25%)	
QC	1.80% (1.41–2.37%)	
NB/NS/PE/NL	2.39% (1.58–3.64%)	
People born after 1964		
BC	0.53% (0.41–0.71%)	
AB/SK/MB	0.46% (0.37–0.56%)	
ON	0.52% (0.47–0.60%)	
QC	0.62% (0.47–0.77%)	
NB/NS/PE/NL	0.77% (0.59–0.97%)	
Proportion of CHC undiagnosed		Hamadeh et al.^8,31,32^
People born before 1945		
BC	32.6% (23.0–41.5%)	
AB/SK/MB	32.5% (18.6–49.7%)	
ON	24.0% (16.4–29.4%)	
QC	19.3% (10.9–30.5%)	
NB/NS/PE/NL	20.4% (10.1–35.6%)	
People born between 1945 and 1964		
BC	21.2% (13.3–29.9%)	
AB/SK/MB	25.7% (18.0–36.7%)	
ON	21.1% (17.6–24.0%)	
QC	30.1% (20.7–40.5%)	
NB/NS/PE/NL	22.7% (10.9–37.8%)	
People born after 1964		
BC	38.8% (26.3–52.7%)	
AB/SK/MB	34.4% (24.9–44.1%)	
ON	44.0% (36.8–48.2%)	
QC	44.4% (31.2–55.6%)	
NB/NS/PE/NL	46.5% (35.6–55.6%)	
Annual probability of F0 progressing to F1	0.107 (0.097–0.118)	Erman et al.^21^
Annual probability of F1 progressing to F2	0.082 (0.074–0.091)	
Annual probability of F2 progressing to F3	0.117 (0.107–0.129)	
Annual probability of F3 progressing to F4	0.116 (0.104–0.131)	
Probability of receiving HCV treatment 6 months after diagnosis	0.95 (0.71–1)	Expert opinion
Probability of receiving second line HCV treatment	0.5 (0.375–0.625)	
HCV treatment efficacy	0.95 (0.935–0.987)	Feld et al., Foster et al., Luo et al.^33–35^
Annual probability of dying from DC	0.216 (0.162–0.27)	D’Amico et al.^25^
Annual probability of dying from HCC	0.411 (0.31–0.51)	Altekruse et al.^36^
Annual probability of dying for general population	Varied by age; see Table S1.3	Statistics Canada^39^
Annual probability of dying in the first year after liver transplant	0.142 (0.124–0.159)	Charlton et al.^37^
Annual probability of dying after the first year of liver transplant	0.034 (0.024–0.043)	
Probability of receiving an antibody test if HCV+ (status quo)	Varied by age; see Table S1.4	Estimation
Probability of receiving an antibody test if HCV− (status quo)		
Relative risk of receiving antibody test if screening recommendations put in place	1.49 (1.12–1.86)	Barocas et al.^38^
Probability of individual following up for an RNA test if antibody test is + ve	0.78 (0.59–0.98)	Janjua et al.^27^
Probability of following up for diagnosis if RNA test is + ve	0.84 (0.63–1)	
Annual probability of reinfection (under 50 years old)	0.02 (0.015–0.025)	Grady et al.^30^
Annual probability of first time HCV infection (under 50 years old)	0.00054 (0.000405–0.000675)	PHAC^27^

### Economics assumptions

This evaluation was conducted from the Canadian provincial payer perspective and structured as a cost–utility analysis and a budget impact analysis. For the cost-utility analysis, costs and benefits were discounted at 1.5% and we used a lifetime time-horizon^39^, while for the budget impact analysis, no discounting was used as per best practice recommendations^40^ and we adopted a 10-year time horizon.

### Analytical strategy and scenario analysis

We first conducted a base-case analysis to estimate the expected value using deterministic calculations. We then ran probabilistic sensitivity analyses (PSA) using the Monte Carlo simulation for 1000 iterations for each sub-group analysis. All probabilistic parameters and utilities used in the model were represented by beta distributions formed by the corresponding ranges (95% confidence interval if available or ± 25% if not available) (Table 1, Supplementary Information Table S1.1–S1.6); all the cost parameters were represented by gamma distributions formed by the corresponding ranges (see Supplementary Information Table S1.9).

We also consider 7 different future scenarios, in particular, the Cancer screening agency model of care, where we assume that a provincial agency exclusively for HCV screening is established, akin to that seen in cancer screening. Such an agency would have additional operating costs and would increase screening rates in all birth cohorts. We also conducted a threshold analysis to understand the minimum prevalence that can make the one-time “case-finding” screening intervention cost-effective. Detail of other scenario analyses and threshold analysis were described in Supplementary Information S2. All methods were carried out in accordance with the Canadian economic evaluation guideline^39^.

### Ethical statement

This project has been approved by the Research Ethics Board at the University Health Network. In addition, this project used selected data from ICES. ICES is a prescribed entity under Ontario’s Personal Health Information Protection Act (PHIPA). Section 45 of PHIPA authorizes ICES to collect personal health information, without consent, for the purpose of analysis or compiling statistical information with respect to the management of, evaluation or monitoring of, the allocation of resources to or planning for all or part of the health system. Projects that use data collected by ICES under section 45 of PHIPA. The use of the data in this project is authorized under section 45 and approved by ICES’ Privacy and Legal Office.

### Patient and public involvement statement

Patients, the public, communities, and stakeholders were not involved in the study.

## Results

### Cost-effectiveness analysis

Table 2 displays the deterministic incremental costs and quality-adjusted-life-years (QALY) of pursuing a one-time HCV screening strategy for different birth cohorts across 10 Canadian provinces. Screening individuals born after 1964 is associated with an incremental cost-effectiveness ratio (ICER) that varies from $27,422/QALY to $42,191/QALY. Therefore, screening these individuals would be considered cost-effective at a $50,000 per QALY willingness to pay threshold. Screening individuals born between 1945–64, the ICER varies from $35,217/QALY to $48,197/QALY. Screening individuals born before 1945 is associated with higher ICER with ICERs ranging from $142,182/QALY to 178,195/QALY. Screening individuals born before 1945 is not cost-effective in any province. The additional cost is not justified by the small additional health gains using a $50,000 per QALY threshold. Table 3 provides further insights into the factors driving the additional health gains associated with each strategy by detailing the number of DC and HCC cases prevented due to the one-time HCV birth cohort screening. Early detection of HCV by screening potentially prevents advanced liver diseases, such as DC and HCC, which can result in significant health losses in terms of both quality and length of life lived.

**Table 2:** Incremental costs and QALYs for HCV birth cohort screening across 10 Canadian provinces.

	Cost (risk-based screening (status quo))	Cost (one-time birth cohort screening)	∆ (cost)	QALYs (risk-based screening (status quo))	QALYs (one-time birth cohort screening)	∆ (QALYs)	ICER	Probability of being cost-effective at WTP of $50,000/QALY (%)
Birth cohort: after 1964								
AB	$321,031	$321,085	$53.80	25.3518	25.3530	0.0013	$42,191	86.7
BC	$210,790	$210,842	$51.90	25.3467	25.3485	0.0018	$29,059	100
MB	$278,385	$278,434	$49.49	25.3518	25.3530	0.0013	$38,811	95.5
NB	$229,716	$229,771	$55.12	25.3453	25.3471	0.0018	$30,147	100
NL	$303,393	$303,455	$62.27	25.3453	25.3471	0.0018	$34,058	99.7
NS	$247,126	$247,183	$56.95	25.3453	25.3471	0.0018	$31,147	100
ON	$221,862	$221,915	$52.49	25.3477	25.3495	0.0018	$29,591	100
PE	$256,275	$256,330	$55.36	25.3453	25.3471	0.0018	$30,277	100
QC	$223,258	$223,312	$54.54	25.3448	25.3468	0.0020	$27,422	100
SK	$267,864	$267,915	$50.23	25.3518	25.3530	0.0013	$39,385	94.4
Birth cohort: 1945–1964								
AB	$347,020	$347,082	$62.61	15.6434	15.6447	0.0013	$46,723	85.7
BC	$216,628	$216,665	$37.11	15.6455	15.6466	0.0011	$35,217	97.8
MB	$301,468	$301,523	$55.90	15.6434	15.6447	0.0013	$41,718	96.2
NB	$249,409	$249,458	$48.71	15.6417	15.6430	0.0012	$39,354	97.6
NL	$336,520	$336,579	$59.66	15.6417	15.6430	0.0012	$48,197	79.6
NS	$269,505	$269,557	$51.98	15.6417	15.6430	0.0012	$41,998	93.9
ON	$239,907	$239,944	$37.59	15.6497	15.6507	0.0009	$39,816	98.0
PE	$293,329	$293,387	$58.05	15.6417	15.6430	0.0012	$46,901	85.5
QC	$247,525	$247,571	$45.63	15.6513	15.6525	0.0012	$37,424	98.9
SK	$281,394	$281,449	$55.13	15.6434	15.6447	0.0013	$41,145	96.5
Birth cohort: before 1945								
AB	$343,298	$343,319	$21.71	6.1770	6.1771	0.0001	$178,195	0
BC	$188,203	$188,211	$8.32	6.1785	6.1786	0.0001	$142,182	0
MB	$297,845	$297,866	$20.07	6.1770	6.1771	0.0001	$164,729	0
NB	$232,557	$232,573	$15.49	6.1768	6.1769	0.0001	$152,864	0
NL	$346,199	$346,217	$18.01	6.1768	6.1769	0.0001	$177,773	0
NS	$267,043	$267,059	$16.29	6.1768	6.1769	0.0001	$160,802	0
ON	$222,449	$222,456	$6.39	6.1786	6.1787	0.0000	$143,220	0
PE	$325,013	$325,031	$17.86	6.1768	6.1769	0.0001	$176,219	0
QC	$243,244	$243,251	$7.31	6.1785	6.1786	0.0000	$154,960	0
SK	$269,985	$270,005	$20.01	6.1770	6.1771	0.0001	$164,187	0

Cost and QALY are expressed in average total per person over the lifetime horizon.

AB, Alberta; BC, British Columbia; MB, Manitoba; NB, New Brunswick; NL, Newfoundland & Labrador; NS, Nova Scotia; ON, Ontario; PE, Prince Edward Island; QC, Quebec; SK, Saskatchewan; ∆, difference; QALY, Quality adjusted Life Years; ICER, Incremental Cost Effectiveness Ratio;

**Table 3:** Number of decompensated cirrhosis (DC) and hepatocellular carcinoma (HCC) cases per province for each birth cohort screening strategy.

	DC cases (risk-based screening (status quo)))	DC cases (one-time birth cohort screening)	∆ (DC cases)	HCC cases (risk-based screening (status quo)))	HCC cases (one-time birth cohort screening)	∆ (HCC cases)
Birth cohort: after 1964						
AB	314	159	155	255	140	115
BC	614	395	219	514	361	153
MB	90	46	44	73	40	33
NB	74	40	33	60	36	25
NL	52	28	24	43	25	17
NS	92	50	42	75	45	31
ON	1740	1106	633	1452	1010	441
PE	14	8	6	12	7	5
QC	1134	735	399	951	673	277
SK	77	39	38	63	34	28
Birth cohort: 1945–1964						
AB	783	652	131	592	518	74
BC	782	639	143	590	506	84
MB	260	216	43	196	172	25
NB	179	149	30	135	118	17
NL	126	106	21	96	84	12
NS	219	183	36	166	145	21
ON	2172	1817	355	1640	1444	195
PE	33	28	5	25	22	3
QC	1629	1344	285	1232	1066	165
SK	225	187	38	170	148	21
Birth cohort: before 1945						
AB	167	157	10	116	111	5
BC	137	129	8	96	91	5
MB	68	64	4	48	45	2
NB	47	44	3	33	31	1
NL	30	28	2	21	20	1
NS	58	55	3	41	39	2
ON	316	299	18	221	211	10
PE	9	8	0	6	6	0
QC	205	193	12	143	136	7
SK	61	57	4	42	40	2

AB, Alberta; BC, British Columbia; MB, Manitoba; NB, New Brunswick; NL, Newfoundland & Labrador; NS, Nova Scotia; ON, Ontario; PE, Prince Edward Island; QC, Quebec; SK, Saskatchewan; ∆, difference.

At a $50,000 per QALY willingness-to-pay threshold, probabilistic sensitivity analysis (Table 1) shows that in over 90% iterations, screening adults born after 1964 produces incremental costs and effects that are cost-effective in all provinces; in over 80% iterations, screening adults born 1945–1964 produces incremental costs and effects that are cost-effective across all provinces. In all (100%) iterations, screening adults born before 1945 produces incremental costs and effects that are not cost-effective for all 10 provinces.

Scenario analyses (Supplementary Information S2) reveals that: assuming a HCV screening program is set up in Ontario at a cost of $2.03 million a year; and a further 16% increase in screening uptake based on evidence from colorectal cancer screening^41^; the incremental cost is higher but screening both cohorts born after 1945 remains cost-effective.

### Budget-impact analysis

Table 4 shows the cumulative net budget impact from HCV screening on each province over a 10-year period. In every province, one-time HCV birth cohort screening leads to a net increase in healthcare costs over ten years meaning that it is not cost saving over this time period. In the short term, curing HCV leads to a large budget impact as treatment costs have to be paid up-front and therefore a diagnosis of HCV leads to higher healthcare costs. In the long run, curing HCV prevents DC and HCC and saves the health system considerable amount of money and thereby reduces the budget impact of screening in the future. The difference in total budget impact across provinces is related to the size of the population being screened and the prevalence of HCV. The budget impact is highest for the youngest cohort. This is partly because this cohort has the largest population, double that of the other two birth cohorts. Secondly, new HCV infections are still likely occurring in this cohort.

**Table 4:** Cumulative net budget impact from HCV birth cohort screening on each province over a 10-year period.

Province	Population size	Net budget impact
Screening birth cohort: after 1964		
AB	2,099,424	$78,648,704
BC	2,216,622	$80,180,984
MB	601,875	$21,828,325
NB	322,030	$14,853,311
NL	227,993	$10,871,616
NS	401,427	$18,683,248
ON	6,395,336	$236,187,349
PE	61,470	$2,776,810
QC	3,640,893	$140,908,968
SK	517,454	$19,210,067
Screening birth cohort: 1945–1964		
AB	954,962	$22,913,869
BC	1,328,001	$29,616,954
MB	316,516	$7,554,160
NB	233,353	$5,273,472
NL	165,137	$3,762,147
NS	285,935	$6,463,108
ON	3,619,439	$61,513,127
PE	43,202	$970,865
QC	2,298,136	$53,626,065
SK	273,930	$6,519,459
Screening birth cohort: before 1945		
AB	315,558	$5,702,800
BC	538,475	$3,922,393
MB	129,202	$2,200,035
NB	93,295	$1,208,087
NL	59,858	$862,065
NS	115,687	$1,551,542
ON	1,472,090	$7,988,890
PE	17,167	$245,324
QC	958,718	$6,055,530
SK	114,610	$1,933,710

AB, Alberta; BC, British Columbia; MB, Manitoba; NB, New Brunswick; NL, Newfoundland & Labrador; NS, Nova Scotia; ON, Ontario; PE, Prince Edward Island; QC, Quebec; SK, Saskatchewan.

Looking at the breakdown of the budget impact, 88% is attributable to DAA drug costs and only 10% related to screening costs, with the remainder contributed by other HCV-related medical costs. The biggest effect on budget impact is the screening uptake, the number of extra people receiving an antibody test and the number of extra diagnoses due to screening. Treatment cost has the second largest effect on the budget impact followed by HCV incidence.

## Discussion

Our analysis shows that one-time HCV screening in individuals born after 1945 is likely a cost-effective use of healthcare resources at a $50,000 per QALY willingness-to-pay threshold. Although current practice is successfully identifying HCV cases, the incremental benefit of a broader, birth-cohort based one-time screening program appears to justify the additional cost. On the other hand, for those born before 1945, our analysis shows that one-time HCV screening only leads to very marginal health gains due to the limited capacity to benefit from a HCV cure.

Cost-effectiveness is only one component that decision makers need to consider when recommending screening policies. Another important consideration is the budget impact. This study shows that, across the ten provinces, screening people born after 1964 would add to the healthcare budget between $2.5 million in PEI and $236 million in Ontario over the next 10 years. Screening baby boomers only would add $1 million (in PEI) to $61 million (in Ontario) over the next 10 years. Screening people born before 1945 would add far less at $250,000 to $8 million over the next 10 years. The size of the budget impact is largely determined by the size of the population with unknown CHC. The size of the unknown CHC population is influenced by: the size of the population, CHC prevalence and the proportion of unknown CHC cases. The more unknown CHC cases there are the larger the incremental impact of screening. Over 10 years, the budget impact for screening baby boomers is relatively small in comparison to the other recently recommended health services and technology by the Ontario Health Technology Assessment Committee of Ontario Health, such as psychotherapy for major depressive disorder and generalized anxiety disorder provided by non-physicians, which has a 5-year budget impact of as much as $329 million for Ontario^17^.

### Comparison with previous studies

Our results are in line with other studies that have attempted to assess the cost-effectiveness of one-time HCV screening among birth cohorts and the general population. Studies by Wong et al. and Eckman et al. supported the notion that one-time screening individuals over age 18 is a cost-effective use of healthcare resources^16,42^. The majority of studies in the literature also supported one-time HCV screening for the general population or the baby boomer cohort^23^.

### Strengths and limitations

This study adds to the growing literature of modeling by combining data sources from systematic reviews, meta-analyses and administrative data to inform key model parameters. This is the first model to assess HCV screening across the 10 Canadian provinces taking into account differences in costs and population characteristics. Although this study uses the best available evidence, there are limitations. First of all, it is not known what an one-time HCV birth cohort screening program actually looks like in the Canadian context. Our analysis adopted two approaches: in the base-case, we assumed the one-time HCV birth cohort screening came from federal and provincial recommendations and clinicians therefore invited more patients to come forward for screening. In our scenario analysis, we assumed that additional costs would be incurred to bring people forward for screening through the use of a provincial HCV screening program. The impact of either approach on screening rates is largely informed by data from the US and limited to baby boomers. However, the full impact of these assumptions on cost-effectiveness has been extensively explored in our scenario analyses. Lastly, although our analysis concluded that one-time screening of selected birth cohorts can be cost-effective, however, the birth cohort composition may change significantly due to the continuous effort on linkage to care and treatment overtime, future updated and expanded analyses will be necessary.

### Policy implications

Over time, individuals with unknown CHC will be harder to find as those who are easy to identify and treat will have already been screened. One potential way to screen the harder to reach individuals might be to employ a screening program dedicated to improve HCV diagnosis rates, akin to that used in cancer screening. Setting up such a program in Ontario at an expense of $2 million a year is considered a cost-effective strategy as long as antibody testing rates increased by more than 10%, relative to the current rates. From a health economic perspective, our scenario analysis shows that a dedicated HCV screening provincial program is of beneficial value. The efficacy and viability of such an option should be considered by Canadian provinces.

## Conclusion

One time birth cohort HCV screening for people born after 1945 is a cost-effective use of healthcare resources in all Canadian provinces. This can be achieved at a significant but still manageable cost to provincial healthcare budgets. We believe our results will complement the clinical, patient preference, feasibility, and equity data that policy decision makers need to consider in developing HCV disease control strategies in the coming decade.

### Supplementary Information

Supplementary Information 1.

Supplementary Information 2.

## Data Availability

All input data for the decision analytical model are available within the article and the supplementary information. The decision analytical model is available from the corresponding author on reasonable request.
