# Supplementary material for: A province-by-province cost-effectiveness analysis and budget impact analysis of one-time birth cohort screening of hepatitis C virus (HCV) infection in Canada
Source: Sci Rep. 2023 Aug 18;13:13484. doi: 10.1038/s41598-023-39521-8 (PMC10439170; doi:10.1038/s41598-023-39521-8)
Supplement: Supplementary file 1 — Supplementary Information 1. [file 41598_2023_39521_MOESM1_ESM.docx]

**A province-by-province cost-effectiveness analysis and budget impact analysis of one-time birth cohort screening of hepatitis C virus (HCV) infection in Canada**

**Supplementary Information S1: Detailed methodology**

# William W. L. Wong^1,2^ PhD | Alex Haines^2^ MSc | Josephine Wong^2^ MD | Abdullah Hamadeh^1^ PhD | Murray D. Krahn^2^ MD, MSc

## We developed a state-transition model of HCV to assess the cost-effectiveness and budget impact of three one-time birth-cohort HCV screening strategies for each of the ten Canadian provinces. Detailed methodology is presented in the following subsections.

## S1.1 Model structure and Study cohort

We constructed a state-based transition model [1] with health states that reflected the natural history of CHC from acute infection to end stage liver disease. This model simulated the transition through health states over time for a given cohort using monthly time intervals. TREEAGE PRO 2018 [2] was used to construct the model.

In the model, the cohort being simulated consisted of all individuals who met the criteria for one-time birth cohort HCV screening. There were three groups within this cohort: those with chronic hepatitis C infection (CHC), those who never had HCV and those who had spontaneous clearance of HCV.

*Individuals without HCV infection*

For those who never contracted HCV, there is a probability that they may be tested for HCV in every cycle. As the HCV antibody test will be negative, they will not receive a follow-up HCV RNA confirmation test. Their cost and health outcomes mirror that of the general population being screened. For example, in the one-time birth cohort screening strategy that targeted baby boomers, costs and health outcomes (utilities and mortality) reflect those of the general baby boomer population. The model also assumed that there is a probability in every cycle for individuals under the age of 50 to contract HCV. These newly infected individuals will enter the acute HCV health state and may develop CHC.

*Individuals with spontaneously cleared HCV*

These individuals will have a positive HCV antibody test and will receive a follow-up RNA test, which is expected to be negative. Apart from the additional cost associated with RNA testing, we assumed that the cost and health outcomes of this group mirror those of individuals who spontaneously clear in the general population.

#####

##### Figure S1.1: Disease progression for those with HCV


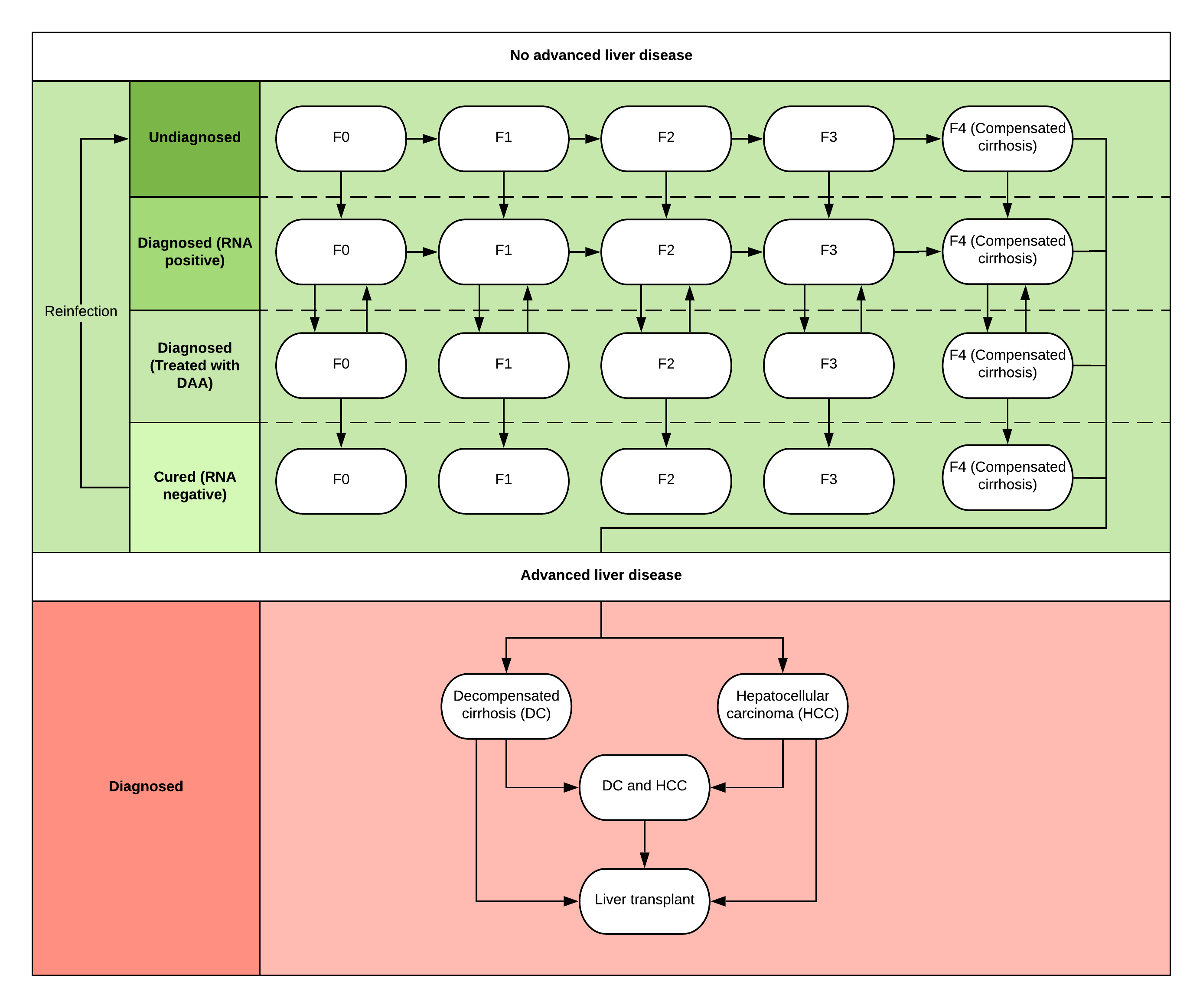


*Individuals with CHC*

Outcomes for those who had CHC were represented in a disease history model outlined in Figure S1.1. When hepatitis C progressed to the chronic stage, the liver began to develop fibrosis, represented by the METAVIR fibrosis stage in our model. Each METAVIR stage (F0, F1, F2, F3, F4) was captured as a separate state in the model. Assuming no prior liver-related disease, the individual started with a fibrosis score of F0. If HCV was left untreated, the liver might continue to scar and the fibrosis score progressed through the 5 stages (F0, F1, F2, F3, F4) until advanced liver disease occurred. At F4 stage, also known as compensated cirrhosis (CC), the individual were at risk of developing advanced liver disease such as decompensated cirrhosis (DC) and/or hepatocellular carcinoma (HCC). Once F4 was reached, the model assumed that an individual could only develop either HCC or DC. Following the development of HCC or DC, individuals were then at risk of developing both conditions or undergoing a liver transplant. After a liver transplant, it was assumed the individual remained in that state until they died. From F0 to F4, CHC could potentially go undiagnosed and silently advance. However, once it progressed to HCC or DC, the assumption was that CHC would be recognized and diagnosed.

*Diagnosing HCV*

The model allowed for individuals to receive an HCV diagnosis at any point. The probability of HCV diagnosis depended on the screening policies in place. If HCV remained undiagnosed, there was a chance in each cycle that individuals would undergo an antibody test. In the one-time birth cohort screening comparators, this probability was higher. Upon obtaining a positive antibody test result, a followed up RNA test was conducted for confirmation. If the RNA test was positive, the individual would follow up by a specialist. At any point after the antibody test, individuals could be lost to follow-up and remain undiagnosed until they underwent another antibody test. The model assumed that individuals with advanced liver disease would have their HCV diagnosed.

*Treating HCV*

Once diagnosed with HCV, the individuals entered a new state with the same fibrosis stage and have a possibility of receiving HCV treatment. While awaiting HCV treatment, they had a chance to progress. Once treatment was administered, the model assumed that fibrosis progression would cease. Patients were considered cured if they achieved a sustained virologic response at 12 weeks post-treatment (SVR12) [3, 4]. Upon being cured, they transitioned to a new state at the same fibrosis stage, which would remain static without further progression. For individuals at fibrosis stage F4, despite being cured of HCV, they still faced the risk of developing advanced liver disease albeit at a significantly reduced rate. If the treatment failed, they might be eligible for second-line therapy, providing another opportunity for a cure. However, if they did not receive second line therapy or if both treatments failed, their fibrosis would continue to advance through the stages.

*Re-infection*

After an individual tested RNA negative, there existed a probability that engaging in high risk activities, such as illicit drug use, might lead to re-infection. In the event of re-infection, the model assumed that the individual would re-enter the undiagnosed HCV state at the fibrosis stage they had achieved when initially cured, with no regression through fibrosis stages. All subsequent probabilities occurred at the same rate as the initial infection. The model assumed that re-infection occurred only once.

The same model structure was used for both the CEA and the BIA.

## S1.2. Probabilities used in the model

*Starting distributions*

To populate the model, data was used to distribute the cohort among the various health states outlined in Figure S1.1.

A back-calculation model was used to calculate the prevalence of CHC and the proportion of undiagnosed cases [5]. It used administrative data from British Columbia and Ontario on the number of diagnosed cases of HCV, HCC and DC to calculate the number of undiagnosed cases of CHC. Details on how this calculation was done can be found in a paper by Hamadeh et al. [6]. We utilized the back-calculation model to determine the distribution of individuals with undiagnosed CHC across the fibrosis stages (F0, F1, F2, F3, F4).

The proportion of people who started in the acute or *spontaneously cleared HCV* state was derived using Public Health of Ontario laboratory data on individuals who had a positive antibody test but a negative RNA test and had not received HCV treatment. The proportion of HCV cases that was found to be acute or *spontaneously cleared HCV* was 17.9% [7, 8, 6].

Individuals who had previously received a diagnosis of CHC were assumed to initiate the model in the 'cured' HCV health state. However, this assumption did not impact the model results since the screening did not affect the cost or health outcomes of individuals already diagnosed with HCV. Among those who were currently diagnosed and cured, it was assumed that 2% would experience re-infection [9, 10]. The remaining portion of the cohort was assigned to the *individuals without HCV infection* state.

All these values were assumed to be province-specific and age-specific and are displayed in Table S1.1 below.

##### Table S1.1: Starting distributions for model by province, separated by age cohort [11]

| Born after 1964 | AB | BC | MB | NB | NL | NS | ON | PE | QC | SK |
| --- | --- | --- | --- | --- | --- | --- | --- | --- | --- | --- |
| Prevalence of CHC | 0.46% | 0.53% | 0.46% | 0.77% | 0.77% | 0.77% | 0.52% | 0.77% | 0.62% | 0.46% |
| Proportion of CHC undiagnosed | 34.4% | 38.8% | 34.4% | 46.5% | 46.5% | 46.5% | 44.0% | 46.5% | 44.4% | 34.4% |
| Proportion undiagnosed (F0) | 52.77% | 17.50% | 52.77% | 48.45% | 48.45% | 48.45% | 25.70% | 48.45% | 23.39% | 52.77% |
| Proportion undiagnosed (F1) | 31.35% | 24.80% | 31.35% | 32.72% | 32.72% | 32.72% | 26.00% | 32.72% | 25.94% | 31.35% |
| Proportion undiagnosed (F2) | 9.78% | 19.20% | 9.78% | 11.20% | 11.20% | 11.20% | 15.60% | 11.20% | 17.14% | 9.78% |
| Proportion undiagnosed (F3) | 3.80% | 17.40% | 3.80% | 4.69% | 4.69% | 4.69% | 13.60% | 4.69% | 15.05% | 3.80% |
| Proportion undiagnosed (F4) | 2.30% | 21.10% | 2.30% | 2.94% | 2.94% | 2.94% | 19.10% | 2.94% | 18.48% | 2.30% |
| Acute HCV | 0.099% | 0.113% | 0.099% | 0.119% | 0.119% | 0.119% | 0.111% | 0.119% | 0.132% | 0.099% |
| Never HCV | 99.44% | 99.36% | 99.44% | 99.32% | 99.32% | 99.32% | 99.37% | 99.32% | 99.25% | 99.44% |
| Born between 1945-64 | AB | BC | MB | NB | NL | NS | ON | PE | QC | SK |
| Prevalence of CHC | 2.24% | 2.31% | 2.24% | 2.39% | 2.39% | 2.39% | 1.93% | 2.39% | 1.80% | 2.24% |
| Proportion of CHC undiagnosed | 25.70% | 21.20% | 25.70% | 22.70% | 22.70% | 22.70% | 21.10% | 22.70% | 30.10% | 25.70% |
| Proportion undiagnosed (F0) | 1.96% | 4.42% | 1.96% | 2.63% | 2.63% | 2.63% | 3.00% | 2.63% | 2.60% | 1.96% |
| Proportion undiagnosed (F1) | 13.11% | 21.20% | 13.11% | 14.96% | 14.96% | 14.96% | 13.30% | 14.96% | 16.48% | 13.11% |
| Proportion undiagnosed (F2) | 16.92% | 20.20% | 16.92% | 16.70% | 16.70% | 16.70% | 14.40% | 16.70% | 19.60% | 16.92% |
| Proportion undiagnosed (F3) | 23.08% | 21.30% | 23.08% | 19.32% | 19.32% | 19.32% | 19.40% | 19.32% | 23.03% | 23.08% |
| Proportion undiagnosed (F4) | 44.93% | 32.90% | 44.93% | 46.39% | 46.39% | 46.39% | 49.80% | 46.39% | 38.29% | 44.93% |
| Acute HCV | 0.70% | 0.77% | 0.70% | 0.59% | 0.59% | 0.59% | 0.64% | 0.59% | 0.56% | 0.70% |
| Never HCV | 97.18% | 96.92% | 97.18% | 97.64% | 97.64% | 97.64% | 97.43% | 97.64% | 97.77% | 97.18% |
| Born before 1945 | AB | BC | MB | NB | NL | NS | ON | PE | QC | SK |
| Prevalence of CHC | 1.60% | 0.75% | 1.60% | 2.09% | 2.09% | 2.09% | 0.75% | 2.09% | 1.03% | 1.60% |
| Proportion of CHC undiagnosed | 32.50% | 32.60% | 32.50% | 20.40% | 20.40% | 20.40% | 24.00% | 20.40% | 19.30% | 32.50% |
| Proportion undiagnosed (F0) | 7.26% | 8.40% | 7.26% | 6.38% | 6.38% | 6.38% | 3.20% | 6.38% | 4.35% | 7.26% |
| Proportion undiagnosed (F1) | 31.35% | 21.20% | 13.11% | 14.96% | 14.96% | 14.96% | 13.30% | 14.96% | 16.48% | 13.11% |
| Proportion undiagnosed (F2) | 9.78% | 20.20% | 16.92% | 16.70% | 16.70% | 16.70% | 14.40% | 16.70% | 19.60% | 16.92% |
| Proportion undiagnosed (F3) | 13.52% | 21.30% | 23.08% | 19.32% | 19.32% | 19.32% | 19.40% | 19.32% | 23.03% | 23.08% |
| Proportion undiagnosed (F4) | 50.29% | 32.90% | 44.93% | 46.39% | 46.39% | 46.39% | 49.80% | 46.39% | 38.29% | 44.93% |
| Acute HCV | 0.274% | 0.161% | 0.274% | 0.428% | 0.428% | 0.428% | 0.161% | 0.428% | 0.284% | 0.274% |
| Never HCV | 98.45% | 99.09% | 98.45% | 97.58% | 97.58% | 97.58% | 99.09% | 97.58% | 98.39% | 98.45% |

*Abbreviations: AB: Alberta; BC: British Columbia; MB: Manitoba; NB: New Brunswick; NL: Newfoundland and Labrador; NS: Nova Scotia; ON: Ontario; PE: Prince Edward Island; QC: Quebec; SK: Saskatchewan; CHC: chronic hepatitis C; HCV: hepatitis C virus; U: Undiagnosed.*

*Probabilities associated with disease progression*

The probabilities used to inform fibrosis progression were taken from an updated systematic review by Erman et al. [12] and are displayed in Table S1.2 below.

##### Table S1.2: Annual fibrosis progression rates

| F0 to F1 | F1 to F2 | F2 to F3 | F3 to F4 |
| --- | --- | --- | --- |
| 0.107 (0.097-0.118) | 0.082 (0.074-0.091) | 0.117 (0.107-0.129) | 0.116 (0.104-0.131) |

Once an individual reached F4 (CC), the probability of developing DC or HCC was contingent on whether the individual had been successfully treated (SVR). For individuals that remained RNA positive, the annual probability used in the model for developing HCC or DC was 2.4% and 3.5%, respectively [13, 14]. For individuals who achieved SVR, the probability of developing HCC or DC fell to 0.5% and 0.2%, respectively. The annual probability used in the model for receiving a liver transplant was 3.3% [15]. These probabilities were assumed to be constant over time.

*Probabilities associated with treatment*

We incorporated a time lag between the time of diagnosis and the initiation of treatment. According to our assumption, 95% of individual diagnosed would have received treatment after a period of 6 months [16].

To calculate treatment efficacy, we used data from ASTRAL studies [17, 18]. In our model, it was assumed that after completing a full course of treatment, 95% of individuals would achieve sustained virologic response (SVR). For those who did not attain SVR, 50% were assumed to receive second-line treatment with comparable efficacy [19]. However, individuals who failed to achieve SVR even after second-line treatment were assumed to have persistent, uncured HCV for the remainder of their lives.

*Probabilities associated with mortality*

Mortality rates were taken from Canadian life tables published by Statistics Canada [20]. These life tables detailed the probability of death for every year of life up to 110 years of age. Table S1.3 below shows how this probability changes over time for each decade of life.

##### Table S1.3: Annual probability of death by age

| Age | Annual, all cause, population-based probability of death |
| --- | --- |
| 20 years | 0.00049 |
| 30 years | 0.00072 |
| 40 years | 0.00104 |
| 50 years | 0.00243 |
| 60 years | 0.00583 |
| 70 years | 0.01498 |
| 80 years | 0.04128 |
| 90 years | 0.12202 |
| 100 years | 0.30486 |

The annual probability of death for an individual with DC was assumed to be 21.6% [21]; and for someone with HCC was assumed to be 41.1% [22]. The annual probability of dying from a liver transplant was assumed to be 14.2% in the first year after the transplant and decrease to 3.4% after the first year [23] . The probability of dying with no advanced liver disease was assumed to be the same as the general population.

*Probability of diagnosis (status quo)*

The probability of an individual currently receiving a HCV-antibody test was estimated using ICES administrative data and the back-calculation model [26]. From 2003 to 2014 we obtained data on the number of individuals who received a positive HCV-antibody test in ON for the first time and used the back-calculation model to derive an estimate for the number of unknown cases of HCV over this time period. Using these two numbers, we calculated the probability of an individual who was unaware of their HCV status receiving an antibody test:

$${Prob(Antibody test)}_{HCV+}= \frac{No. {unknown CHC}_{pos} people who receive an antibody test}{No. people with unknown CHC}$$

We also derived an estimate for the probability of receiving a HCV-antibody test for those without HCV. A study from BC showed that 5.6% of HCV-antibody tests came back positive [24]. This meant for every 56 tests per 1000 tests that came back positive, 944 came back negative. Based on this ratio, we performed calculations to determine the quantity of antibody tests conducted on an HCV-negative cohort. As an illustration, in 2014, there were 1,729 positive antibody test results in ON for individuals born between 1945 and 1964. Applying the same ratio mentioned above, this would imply that 30,875 tests yielded negative results. Thus, we derived the probability of receiving an antibody test for those without HCV:

$${Prob(Antibody test)}_{HCV-}= \frac{No. {CHC}_{neg} people who receive an antibody test}{No. people without CHC}$$

We calculated these probabilities for the three cohorts: born before 1945, born 1945-1964, born after 1964.

To incorporate these figures into the model, we needed to make an assumption regarding their potential changes over time. While the data indicated a decrease in the annual number of HCV diagnoses, the number of individuals with undiagnosed HCV was also declining due to the low incidence within the baby boomer cohort. Figure S1.2 illustrates the evolving proportion of previously unknown cases that were subsequently diagnosed. These time-series were derived from 1) prevalence and undiagnosed numbers obtained through back-calculation, and 2) diagnosed numbers based on ICES. Across all three birth cohorts, the lines representing these proportions remained relatively stable over time, albeit showing a slight increase in the youngest age cohort in recent years. Consequently, we made the assumption that the proportion of unknown cases being diagnosed would remain constant over time. This meant that the probability of undergoing an antibody test would remain consistent throughout the timeframe considered in the model.

##### Figure S1.2: Proportion of unknown cases of HCV diagnosed over time by age group

##### Table S1.4: Annual probability of diagnosis by age

| \| Age \| Born < 1945 \| Born 1945-64 \| Born after 1964 \| \| --- \| --- \| --- \| --- \| \| Probability of unknown CHC individual receiving antibody test \| 4.03% \| 9.03% \| 12.68% \| \| Probability of HCV negative individual receiving antibody test \| 0.35% \| 0.82% \| 0.46% \| |  |  |  |
| --- | --- | --- | --- | --- | --- | --- | --- | --- | --- | --- | --- | --- | --- | --- | --- |

*Probability of diagnosis (one time birth cohort screening)*

In the case of one-time birth cohort screening, we made the assumption that the rate of individuals receiving an antibody test would follow the same increase observed in the US after the CDC recommended screening baby boomers. A study found that the screening rate in baby boomers increased by 50% in the years after the CDC recommendation. We assumed this rate increase remained constant over time and applied equally to those with or without HCV [25].

*Probability of loss to follow-up*

There were two steps to diagnosing HCV: First, an HCV antibody test to determine whether the individual had been infected followed by an RNA test to confirm. We assumed these tests had perfect diagnostic accuracy and therefore a sensitivity and specificity of 100%. However, we assumed a loss to follow-up from receiving the first antibody test and receiving the HCV diagnosis. The proportion of drop-off was taken from a cascade of care study conducted in BC using administrative data. They found that 22% of people did not return for a follow-up RNA test after a positive antibody test and 16% did not return after a positive RNA test to discuss the diagnosis [7]. For individuals who were not followed-up all the way to diagnosis, we assumed they remained undiagnosed. We also assumed that the probability of loss of follow-up remain unchanged between the two screening interventions.

*Probability of re-infection*

After an individual became RNA negative, there was a probability of re-infection. We derived this probability from a study by Grady et al [10] which found that the reinfection rate was 2.67 per 100 person years. As with most studies on re-infection, they were based on high-risk cohorts that participated in transmission activities.

*Probability of contracting HCV*

During each cycle, there exists the potential for individuals who are HCV-negative to acquire the virus. The probability of this occurrence was determined using data from Remis et al. According to the study, the annual incidence rate of HCV among individuals under the age of 50 was found to be 0.00054 [26]. In the model, we assumed this value remained constant over time.

## S1.3. Utility and cost parameters

### S1.3.1 Utility estimates

The utility associated with each state outlined in Figure S1 was derived from a recent systematic review of all published health state utility values in HCV. [27]. We used the regression model (Table S1.5) reported in Saeed et al. to estimate the health state utility values.

##### Table S1.5: Regression model used to predict utilities in people with HCV

| Variable | Definition | Value (standard error) |
| --- | --- | --- |
| Y | Dependent variable, health state utility values |  |
| β_0_ | Intercept: male; age 51; no cirrhosis; not on treatment; analyzed using EQ-5D-3L. | 0.8334 (0.0516) |
| β_1_X_1_ | Continuous variable; utility changes at a linear rate where X_1_ is the number of years above 51 | -0.0035 (0.0037) |
| β_2_X_2_ | Dummy variable; gender; if female then (X_2_=1) | -0.1511 (0.0962) |
| β_3_X_3_ | Dummy variable; compensated cirrhosis; yes then (X_3_=1) | -0.0577 (0.0235) |
| β_4_X_4_ | Dummy variable; decompensated cirrhosis; yes then (X_4_=1) | -0.0662 (0.0319) |
| β_5_X_5_ | Dummy variable; hepatocellular Carcinoma; yes then (X_5_=1) | -0.0672 (0.0441) |
| β_6_X_6_ | Dummy variable; post-liver transplant; yes then (X_6_=1) | -0.0494 (0.0275) |
| β_7_X_7_ | Dummy variable; on interferon-free treatment; yes then (X_7_=1) | -0.0284 (0.0074) |
| β_8_X_8_ | Dummy variable; sustained virologic response; yes then (X_8_=1) | 0.0375 (0.0045) |

Using the regression model, we estimated the health state utility values for each of the health states (Table S1.6).

##### Table S1.6: Estimates of health state utility values

| Age | No cirrhosis | CC | DC | HCC | DC & HCC | LT | SVR  (no CC) | SVR (CC) |
| --- | --- | --- | --- | --- | --- | --- | --- | --- |
| 20 | 0.9419 | 0.8842 | 0.8757 | 0.8747 | 0.8085 | 0.8925 | 0.9794 | 0.9217 |
| 30 | 0.9069 | 0.8492 | 0.8407 | 0.8397 | 0.7735 | 0.8575 | 0.9444 | 0.8867 |
| 40 | 0.8719 | 0.8142 | 0.8057 | 0.8047 | 0.7385 | 0.8225 | 0.9094 | 0.8517 |
| 50 | 0.8334 | 0.7757 | 0.7672 | 0.7662 | 0.7000 | 0.7840 | 0.8709 | 0.8132 |
| 60 | 0.7984 | 0.7407 | 0.7322 | 0.7312 | 0.6650 | 0.7490 | 0.8359 | 0.7782 |
| 70 | 0.7284 | 0.6707 | 0.6622 | 0.6612 | 0.5950 | 0.6790 | 0.7659 | 0.7082 |
| 80 | 0.6234 | 0.5657 | 0.5572 | 0.5562 | 0.4900 | 0.5740 | 0.6609 | 0.6032 |
| 90 | 0.4834 | 0.4257 | 0.4172 | 0.4162 | 0.3500 | 0.4340 | 0.5209 | 0.4632 |

Abbreviations: CC, compensated cirrhosis; DC, decompensated cirrhosis; HCC, hepatocellular carcinoma; LT, liver transplant; SVR, sustained virologic response.

The following assumptions were made in the model:

- The utility of undiagnosed HCV was the same as diagnosed.
- There was a sustained utility increase from being cured (SVR), as described in the data from Saeed et al.
- While on treatment, the utility decreased by -0.0284 based on the regression model indicated in S1.5. After the completion of treatment, this disutility no longer applied.

### S1.3.2 Costs

The cost associated with each health state was calculated using Ontario population level administrative data held at the ICES, formally known as Institute for Clinical Evaluative Sciences. ICES is an independent, non-profit research institute whose legal status under Ontario’s health information privacy law allows it to collect and analyze health care and demographic data, without consent, for health system evaluation and improvement. We used standard costing methods for administrative data to assign cost estimates to resources used [28]. Individuals in ON were identified as having HCV from 2003 to 2014 using linked data from Public Health Ontario on HCV antibody and RNA tests. Once identified as having HCV, individuals were followed up and their healthcare costs were tracked from the index date (date of positive antibody test) until December 30, 2016 unless they either: died, lost OHIP eligibility or reached 106 years in age. ICES data contained records related to physician billings, outpatient diagnostic tests, inpatient care, ambulatory care, day surgery, drugs for those >65 years, long-term care, and home care. These datasets were linked using unique encoded identifiers and analyzed at ICES. The perspective of the analysis was that of the public health care payer. Costs that fell on the patient (out of pocket costs) or on private insurers were excluded.

We developed an algorithm to assign resource consumption and costs to each of the health state in the model.[29] At the index date, an individual was allocated to one of the nine mutually exclusive states. The entry criteria for each state are shown in Table S1.7 below. Definition of each condition was established using administrative, diagnostic, procedure and death codes. The only state the individual could not enter at the index date was “No cirrhosis (RNA negative)”, as only CHC cases were considered. Once allocated to a state the individual remained in that state until the end of follow-up or until they met the criteria for entry into another state.

##### Table S1.7: Health states and entry criteria

| Health state | Entry criteria |
| --- | --- |
| No cirrhosis | Diagnosis of HCV AND criteria for any other state is not met. |
| No cirrhosis (RNA negative) | The individual receives an RNA negative test result and has no liver disease (CC, DC or HCC). |
| Compensated cirrhosis (CC) | The individual is diagnosed with cirrhosis OR,  5 years prior to the day the individual is diagnosed with decompensated cirrhosis. |
| Decompensated cirrhosis (DC) | The individual is diagnosed with decompensated cirrhosis. |
| Hepatocellular carcinoma (HCC) | The individual is diagnosed with hepatocellular carcinoma. |
| Decompensated cirrhosis AND Hepatocellular carcinoma (DC and HCC) | The individual is diagnosed with HCC whilst already having a DC diagnosis OR, the individual is diagnosed with DC whilst already having a HCC diagnosis. |
| Liver transplant (LT) | The individual receives a liver transplant |
| Terminal (liver related) | 6 months prior to the day of death and the individual has advanced liver disease (DC, HCC or LT) |
| Terminal (non-liver related) | 6 months prior to the day of death and the individual has no advanced liver disease |

Figure S1.3 below shows the possible movements between states an individual could take in this costing algorithm.

##### Figure S1.3: Algorithm for movement between states over time used for the costing model


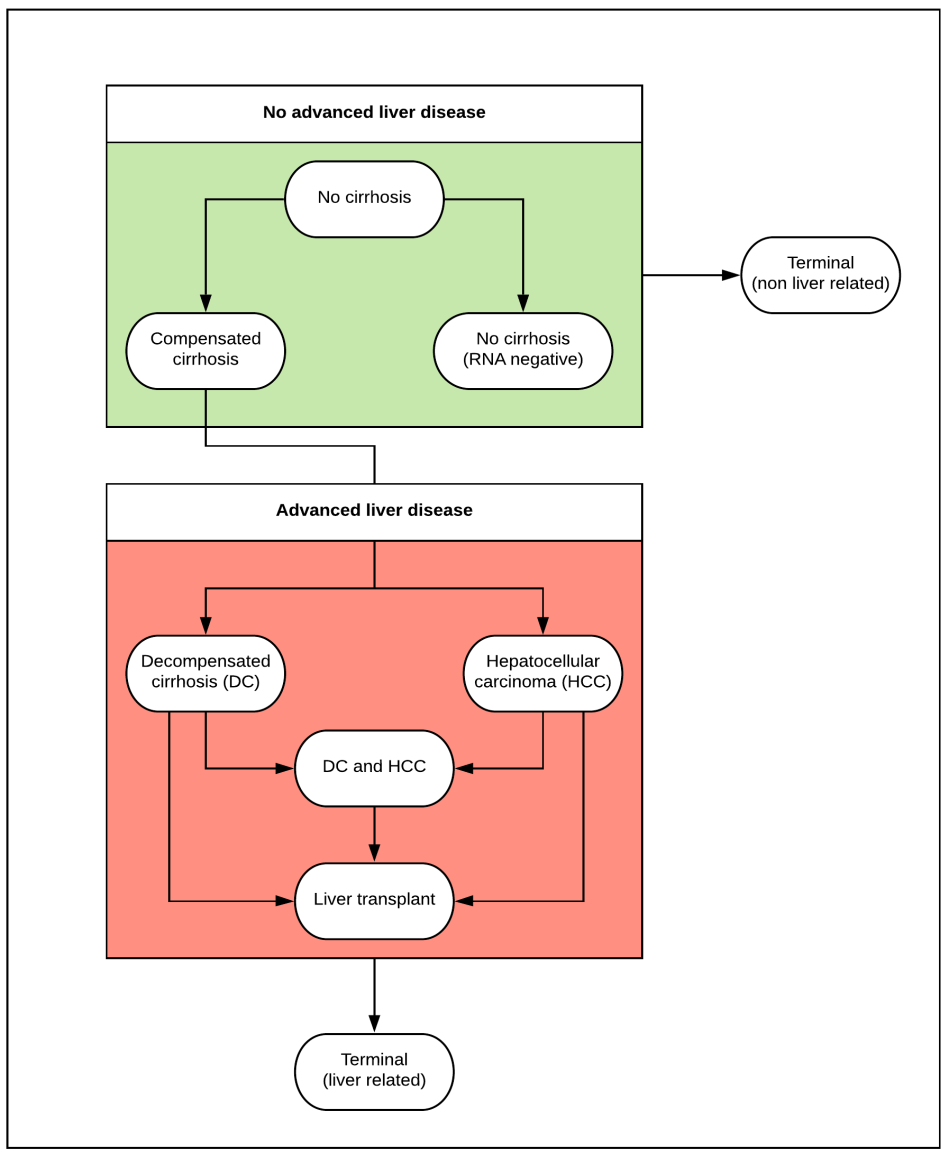


Movement between health states in the costing model was congruent with that in the economic model. However, the costing model assumed that individuals with no cirrhosis and a METAVIR score between F0 and F3 had the same cost, because the administrative data did not have elements that differentiated fibrosis stages.

Once the costs had been collected for each health state, generalized mixed effects regression models were built to predict the 30-day cost based on patient’s baseline characteristics. Covariates included were age, gender, co-morbidity (ADG Score), residence, treatment and immigrant status. Based on the natural history of CHC, four regression models were built: one cost model for individuals who did not have advanced liver disease (DC, HCC, LT); one for those with advanced liver disease; one for individuals in the last 6 months of life; and one for costs that occurred one year prior to a HCV diagnosis in individuals without advanced liver disease. Details of the regression models are presented in Table S1.8 below.

##### Table S1.8: Regression models built for costs

| Variable | Definition |
| --- | --- |
| Y = | Dependent variable, costs per 30 days |
| β_0 +_ | Intercept |
| β_1_X_1 +_ | Age, where (X_1_=Age in years) |
| β_2_X_1_^2^ _+_ | Age^2^, where (X_1_^2^=Age in years squared) to account for non-linear impact of costs on age (*) |
| β_3_X_3 +_ | Dummy variable; Male; yes then (X_3_=1) |
| β_4_X_4 +_ | Dummy variable; Income quintile 2; yes then (X_4_=1) |
| β_5_X_5 +_ | Dummy variable; Income quintile 3; yes then (X_5_=1) |
| β_6_X_6 +_ | Dummy variable; Income quintile 4; yes then (X_6_=1) |
| β_7_X_7 +_ | Dummy variable; Income quintile 5; yes then (X_7_=1) |
| β_8_X_8 +_ | Dummy variable; ADG score (0-3); yes then (X_8_=1) |
| β_9_X_9 +_ | Dummy variable; ADG score (4-7); yes then (X_9_=1) |
| β_10_X_10 +_ | Dummy variable; ADG score (8-10); yes then (X_10_=1) |
| β_11_X_11 +_ | Dummy variable; ADG score (11+); yes then (X_11_=1) |
| β_12_X_12 +_ | Dummy variable; immigrant; yes then (X_12_=1) |
| β_13_X_13 +_ | Dummy variable; received HCV treatment; yes then (X_13_=1) (†) |
| β_14_X_14 +_ | Dummy variable; after three months since entering new health state; yes then (X_15_=1) |
| Variables only used in the ‘no advanced liver’ and ‘one year prior’ disease model | |
| β_15_X_15 +_ | Dummy variable; HCV no cirrhosis; yes then (X_15_=1) |
| β_16_X_16 +_ | Dummy variable; HCV cirrhosis; yes then (X_16_=1) |
| Variables only used in the ‘advanced liver disease model | |
| β_15_X_15 +_ | Dummy variable; HCV and DC; yes then (X_15_=1) |
| β_16_X_16 +_ | Dummy variable; HCV and HCC; yes then (X_16_=1) |
| β_17_X_17 +_ | Dummy variable; HCV and DC and HCC; yes then (X_17_=1) |
| Variables only used in the ‘terminal’ model | |
| β_15_X_15_ | Dummy variable; HCV and dies from liver related disease; yes then (X_15_=1) |

*(†) HCV treatment variable is not used in the costs one year prior model*

*(*) Age^2^ was not used in the advanced liver disease model as no non-linear relationship was detected, meaning costs increased at a linear rate with age.*

The results from the four regressions are outlined in Table S1.9 below. These variables were used to calculate the cost per 30 days for an individual with HCV in a particular state with certain characteristics. The 30-day cost was the exponent of the sum of the applicable variables. Negative variables reduced the cost per 30 days and positive variables increased the cost. For example, an individual diagnosed with HCV but had no CC and was: male, aged 58, ADG score of 7, non-immigrant, and had not received HCV treatment would be assigned the following cost per 30 day period:

$${Cost}_{per 30 days}=\exp\left( 7.7206-0.0576*58+{58}^{2}*0.0007+0.0633-0.0676+0.0917 \right)=\$917$$

##### Table S1.9: Regression model to calculate costs per 30 days for each health state

| State | No advanced liver disease | Advanced liver disease | One year prior to diagnosis | Last six months of life |
| --- | --- | --- | --- | --- |
| β_0_ | 7.7206 | 8.1738 | 6.8856 | 7.8661 |
| β_1_X_1_ (Age in years) | (-)0.0576 | 0.0044 | (-)0.0531 | 0.0247 |
| β_2_X_2_^2^ (Age in years squared) | 0.0007 | - | 0.0006 | (-)0.0002 |
| β_3_X_3_ (Male) | 0.0633 | (-)0.0023 | 0.0570 | (-)0.0635 |
| β_4_X_4_ (Income quintile 2) | (-)0.0676 | (-)0.1158 | (-)0.0931 | 0.0706 |
| β_5_X_5_ (Income quintile 3) | (-)0.1369 | (-)0.0972 | (-)0.1774 | 0.0505 |
| β_6_X_6_ (Income quintile 4) | (-)0.1180 | (-)0.0823 | (-)0.1881 | 0.0380 |
| β_7_X_7_ (Income quintile 5) | (-)0.2376 | (-)0.1724 | (-)0.2325 | (-)0.0157 |
| β_8_X_8_ (ADG 0-3) | (-)0.4389 | 0.0568 | (-)0.8942 | (-)0.2844 |
| β_9_X_9_ (ADG 8-10) | 0.4922 | 0.2535 | 0.7677 | 0.3907 |
| β_10_X_10_ (ADG 11+) | 1.2353 | 0.5825 | 1.6488 | 0.7412 |
| β_11_X_11_ (Immigrant) | (-)0.4778 | (-)0.2333 | (-)0.4682 | 0.1749 |
| β_12_X_12_ (Treatment) | 0.9742 | 0.5793 | - | 0.0639 |
| β_13_X_13_ (no cirrhosis) | 0.0917 | - | - | - |
| β_14_X_14_ (cirrhosis) | 0.4236 | - | (-)0.0545 | - |
| β_15_X_15_ (after 3 months since entry into health state) | (-)0.3711 | (-)0.8925 | - | - |
| β_16_X_16_ (HCC) | - | 0.0933 | - | - |
| β_17_X_17_ (DC & HCC) | - | 0.5238 | - | - |
| β_18_X_18_ (Liver transplant) | - | 0.1162 | - | - |
| β_19_X_19_ (liver related death) | - | - | - | 0.2184 |

To estimate costs for each birth cohort we had to make assumptions about the composition of the cohort. We assumed for all states that: 10% were immigrants, 35% were female and all resided in income quintile 2.

Figure S1.4 below shows how cost varies by age in Ontario. The costs for non-advanced liver disease states were higher in younger cohorts then fell and eventually began to rise around 50 years of age. These increases were in line with how age influenced costs for the general population. For advanced liver disease states, the influence of age on cost was not as dramatic and costs increased with age at a linear rate.

##### Figure S1.4: Costs per 30 days by age (ON)


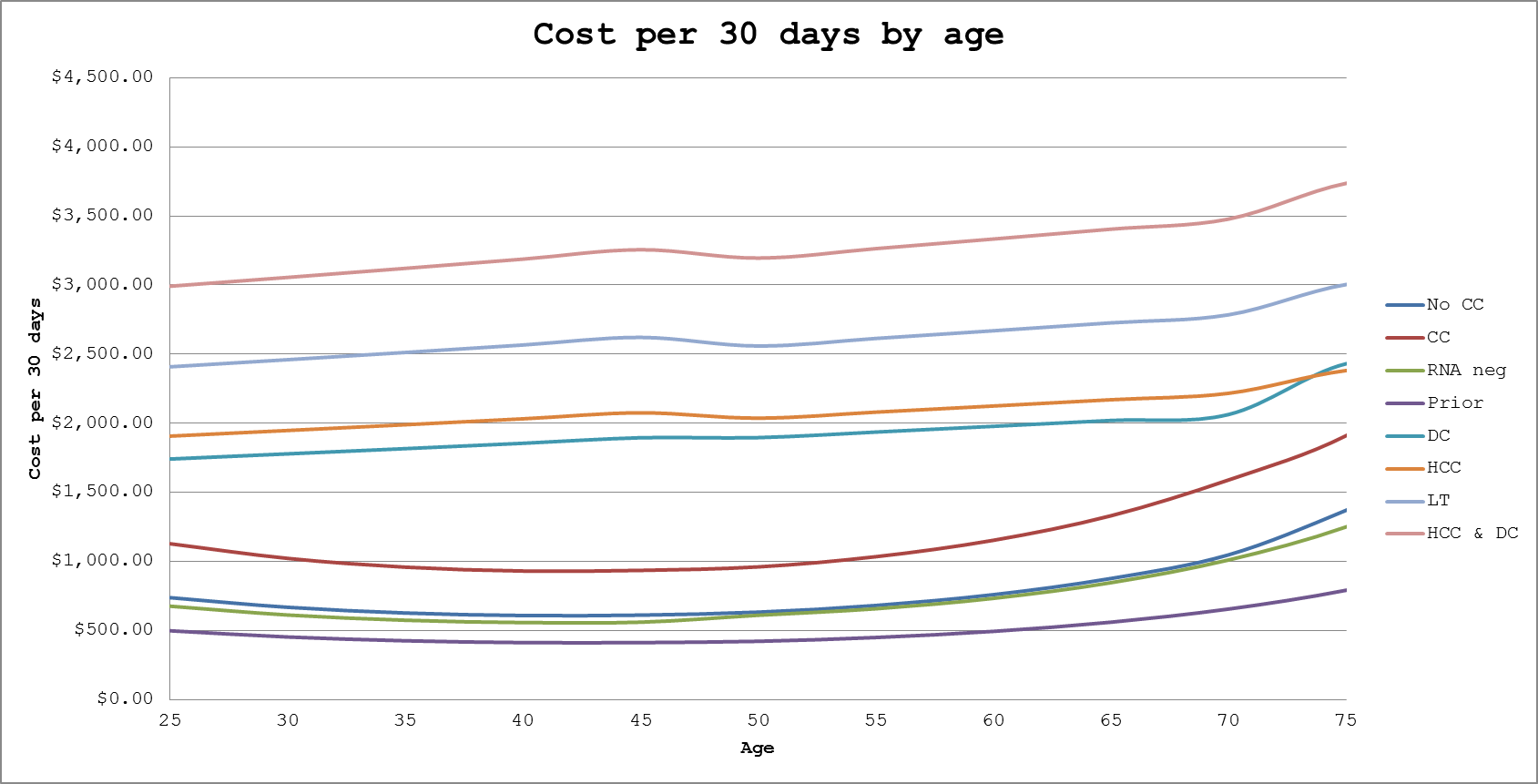


Abbreviations: CC, compensated cirrhosis; RNA neg, ribonucleic acid negative; DC, decompensated cirrhosis; HCC, hepatocellular carcinoma; LT, liver transplant

*Province specific costs*

We used data from the Canadian Institute for Health Information to calculate how costs differed across provinces relative to ON. This adjuster had limitations because it included differences in both patterns of care and unit cost. Taking the age-adjusted per capita spending costs for each province, we calculated how costs differed across provinces relative to ON. In Table S1.10 below the values represented how much higher, or lower, healthcare costs were for each province for a given age bracket, relative to ON.

##### Table S1.10: Regression model to calculate costs per 30 days for each health state

| Age Groups | NL | PE | NS | NB | QC | ON | MB | SK | AB | BC |
| --- | --- | --- | --- | --- | --- | --- | --- | --- | --- | --- |
| 20-24 | 1.432 | 1.084 | 1.279 | 1.127 | 1.062 | 1.000 | 1.403 | 1.626 | 1.582 | 1.283 |
| 25-29 | 1.338 | 1.043 | 1.144 | 1.069 | 1.023 | 1.000 | 1.300 | 1.448 | 1.441 | 1.255 |
| 30-34 | 1.210 | 1.002 | 1.030 | 0.951 | 0.921 | 1.000 | 1.173 | 1.303 | 1.319 | 1.184 |
| 35-39 | 1.284 | 0.903 | 1.029 | 0.961 | 0.891 | 1.000 | 1.221 | 1.320 | 1.372 | 1.167 |
| 40-44 | 1.272 | 0.909 | 1.070 | 1.001 | 0.888 | 1.000 | 1.292 | 1.375 | 1.484 | 1.144 |
| 45-49 | 1.240 | 0.860 | 1.093 | 1.051 | 0.905 | 1.000 | 1.268 | 1.492 | 1.512 | 1.117 |
| 50-54 | 1.210 | 1.000 | 1.085 | 1.005 | 0.926 | 1.000 | 1.246 | 1.301 | 1.425 | 1.112 |
| 55-59 | 1.272 | 0.939 | 1.079 | 1.013 | 0.931 | 1.000 | 1.230 | 1.225 | 1.427 | 1.090 |
| 60-64 | 1.284 | 0.895 | 1.053 | 1.053 | 0.952 | 1.000 | 1.234 | 1.150 | 1.427 | 1.071 |
| 65-69 | 1.183 | 0.910 | 1.012 | 0.996 | 0.926 | 1.000 | 1.135 | 1.112 | 1.271 | 0.939 |
| 70-74 | 1.237 | 0.966 | 1.024 | 1.022 | 0.958 | 1.000 | 1.146 | 1.149 | 1.320 | 0.921 |
| 75-79 | 1.325 | 1.138 | 1.095 | 1.062 | 1.034 | 1.000 | 1.216 | 1.135 | 1.409 | 0.926 |
| 80-84 | 1.450 | 1.410 | 1.187 | 1.059 | 1.104 | 1.000 | 1.280 | 1.143 | 1.508 | 0.901 |
| 85-89 | 1.551 | 1.528 | 1.197 | 1.031 | 0.995 | 1.000 | 1.205 | 1.243 | 1.494 | 0.804 |

*Abbreviations: AB: Alberta; BC: British Columbia; MB: Manitoba; NB: New Brunswick; NL: Newfoundland and Labrador; NS: Nova Scotia; ON: Ontario; PE: Prince Edward Island; QC: Quebec; SK: Saskatchewan*

We applied these multipliers to all state costs used in the model. We assumed the cost of HCV testing was the same across provinces.

*Costs of testing[30]*

The cost of an antibody test was $41.70 which included the cost of test itself ($3), a phlebotomist ($5) and a healthcare provider to prescribe the test and discuss the results ($33.70).

The cost of an RNA test was $113.70 which included the cost of test itself ($75), a phlebotomist ($5) and a healthcare provider to prescribe the test and discuss the results ($33.70).

**References**

1. Siebert U, Alagoz O, Bayoumi AM, Jahn B, Owens DK, Cohen DJ et al. State-transition modeling: a report of the ISPOR-SMDM modeling good research practices task force–3. Medical Decision Making. 2012;32(5):690-700.

2. Erman A, Krahn MD, Hansen T, Wong J, Bielecki JM, Feld JJ et al. Estimation of fibrosis progression rates for chronic hepatitis C: a systematic review and meta-analysis update. BMJ Open. 2019;9(11):e027491. doi:10.1136/bmjopen-2018-027491.

3. Smith-Palmer J, Cerri K, Valentine W. Achieving sustained virologic response in hepatitis C: a systematic review of the clinical, economic and quality of life benefits. BMC infectious diseases. 2015;15(1):19.

4. Puoti M, Foster GR, Wang S, Mutimer D, Gane E, Moreno C et al. High SVR12 with 8-week and 12-week glecaprevir/pibrentasvir therapy: an integrated analysis of HCV genotype 1–6 patients without cirrhosis. Journal of hepatology. 2018;69(2):293-300.

5. THETA Collaborative. Estimation of Hepatitis C Prevalence in Canada (Interim epidemiological report). THETA Collaborative2018.

6. Hamadeh A FZ, Krahn M, Wong W.W.L. . A model-based framework for chronic hepatitis C prevalence estimation. PloSone. 2019;14(11):e0225366-e. doi:10.1371/journal.pone.0225366.

7. Janjua NZ, Kuo M, Yu A, Alvarez M, Wong S, Cook D et al. The population level cascade of care for hepatitis C in British Columbia, Canada: the BC Hepatitis Testers Cohort (BC-HTC). EBioMedicine. 2016;12:189-95.

8. Hamadeh A HA, Feng Z, Thein H, Janjua NZ, Krahn M, Wong W.W.L. Prevalence estimates of chronic hepatitis C in British Columbia and Ontario using health administrative data. . Journal of Hepatology, submitted. 2019.

9. Falade‐Nwulia O, Sulkowski MS, Merkow A, Latkin C, Mehta SH. Understanding and addressing hepatitis C reinfection in the oral direct‐acting antiviral era. Journal of viral hepatitis. 2018;25(3):220-7.

10. Grady BP, Schinkel J, Thomas XV, Dalgard O. Hepatitis C virus reinfection following treatment among people who use drugs. Clinical infectious diseases. 2013;57(suppl_2):S105-S10.

11. Hamadeh A, Feng Z, Krahn M, Wong W. A model-based framework for chronic hepatitis C prevalence estimation. PloSone. 2019;14(11):e0225366-e.

12. Eckman MH, Ward JW, Sherman KE. Cost Effectiveness of Universal Screening for Hepatitis C Virus Infection in the Era of Direct-Acting, Pangenotypic Treatment Regimens. Clin Gastroenterol Hepatol. 2019;17(5):930-9 e9. doi:10.1016/j.cgh.2018.08.080.

13. van der Meer AJ, Veldt BJ, Feld JJ, Wedemeyer H, Dufour J-F, Lammert F et al. Association between sustained virological response and all-cause mortality among patients with chronic hepatitis C and advanced hepatic fibrosis. Jama. 2012;308(24):2584-93.

14. Ng V, Saab S. Effects of a sustained virologic response on outcomes of patients with chronic hepatitis C. Clinical Gastroenterology and Hepatology. 2011;9(11):923-30.

15. Krahn M, Wong JB, Heathcote J, Scully L, Seeff L. Estimating the prognosis of hepatitis C patients infected by transfusion in Canada between 1986 and 1990. Medical Decision Making. 2004;24(1):20-9.

16. Haines A MA, Wong WWL, Krahn M Strategies in achieving population control of HCV infection: Results of a multidisciplinary focus group. . Canadian Liver Journal, revision requested. 2019.

17. Feld JJ, Jacobson IM, Hézode C, Asselah T, Ruane PJ, Gruener N et al. Sofosbuvir and Velpatasvir for HCV Genotype 1, 2, 4, 5, and 6 Infection. New England Journal of Medicine. 2015;373(27):2599-607. doi:10.1056/NEJMoa1512610.

18. Foster GR, Afdhal N, Roberts SK, Bräu N, Gane EJ, Pianko S et al. Sofosbuvir and Velpatasvir for HCV Genotype 2 and 3 Infection. New England Journal of Medicine. 2015;373(27):2608-17. doi:10.1056/NEJMoa1512612.

19. Bourlière M, Gordon SC, Flamm SL, Cooper CL, Ramji A, Tong M et al. Sofosbuvir, Velpatasvir, and Voxilaprevir for Previously Treated HCV Infection. New England Journal of Medicine. 2017;376(22):2134-46. doi:10.1056/NEJMoa1613512.

20. Canadian Agency for Drugs and Technologies in Health. Guidelines for the economic evaluation of health technologies: Canada. 4th edition. In: CADTH, editor. Ottawa2017.

21. D'Amico G, Garcia-Tsao G, Pagliaro L. Natural history and prognostic indicators of survival in cirrhosis: a systematic review of 118 studies. Journal of hepatology. 2006;44(1):217-31.

22. Altekruse SF, McGlynn KA, Reichman ME. Hepatocellular carcinoma incidence, mortality, and survival trends in the United States from 1975 to 2005. Journal of clinical oncology. 2009;27(9):1485.

23. Charlton M, Seaberg E, Wiesner R, Everhart J, Zetterman R, Lake J et al. Predictors of patient and graft survival following liver transplantation for hepatitis C. Hepatology. 1998;28(3):823-30.

24. Janjua NZ, Yu A, Kuo M, Alvarez M, Cook D, Wong J et al. Twin epidemics of new and prevalent hepatitis C infections in Canada: BC Hepatitis Testers Cohort. BMC infectious diseases. 2016;16(1):334.

25. Barocas JA, Wang J, White LF, Tasillo A, Salomon JA, Freedberg KA et al. Hepatitis C testing increased among baby boomers following the 2012 change to CDC testing recommendations. Health Affairs. 2017;36(12):2142-50.

26. Remis R. Modelling the incidence and prevalence of hepatitis C infection and its sequelae in Canada, 2007. Final report. Ottawa (ON): Public Health Agency of Canada; 2007. Available: www phac-aspc gc ca/sti-its-surv-epi/model/pdf/model07-eng pdf (accessed 2015 Jan 16). 2007.

27. Saeed YA PA, Bielecki JM, Mitsakakis N, Bremner KE, Abrahamyan L, Pechlivanoglou P, Feld JJ, Krahn M, Wong WWL. . A Systematic Review and Meta-analysis of Health Utilities in Chronic Hepatitis C Patients. . Value in Health 2020;23(1).

28. Wodchis W BK, Nikitovic M, McKillop I. Guidelines on Person-Level Costing Using Administrative Databases in Ontario. HSPRN Working paper series. Volume 1, May 2013. Health System Performance Research Network. 2013.

29. Wong WWL, Haines A, Bremner K, Zhan Y, Calzavara A, Mitsakakis N et al. Health care costs associated with chronic hepatitis C virus infection: A real-world, population-based analysis in Ontario. CMAJ Open 2020;in press.

30. Mendlowitz A ND, Isaranuwatchai W, Wong W.W.L., Krahn M. An economic evaluation of emergency department population-based hepatitis C screening strategies in Canada. . The 2018 Canadian Liver Meeting. ; Toronto, ON, Canada2018.
