# Supplementary material for: A province-by-province cost-effectiveness analysis and budget impact analysis of one-time birth cohort screening of hepatitis C virus (HCV) infection in Canada
Source: Sci Rep. 2023 Aug 18;13:13484. doi: 10.1038/s41598-023-39521-8 (PMC10439170; doi:10.1038/s41598-023-39521-8)
Supplement: Supplementary file 2 — Supplementary Information 2. [file 41598_2023_39521_MOESM2_ESM.docx]

# **A province-by-province cost-effectiveness analysis and budget impact analysis of one-time birth cohort screening of hepatitis C virus (HCV) infection in Canada**

# **Supplementary Information S2: Scenario Analysis and Threshold Analysis**

# William W. L. Wong^1,2^ PhD | Alex Haines^2^ MSc | Josephine Wong^2^ MD | Abdullah Hamadeh^1^ PhD | Murray D. Krahn^2^ MD, MSc

## S2.1 Scenario analysis

Six different future scenarios were considered:

1. **Improving linkage to care**: Currently, HCV is diagnosed using a two-step testing approach, antibody testing followed by RNA testing. Hepatitis C reflex testing is an alternative that will reduce the loss to follow-up.
2. **Reducing unnecessary re-testing**: Currently, there is no automated system to flag who has received an HCV antibody test. An individual can receive an antibody test multiple times. A scenario was run where an individual with a negative antibody test would be precluded from receiving another test.
3. **Increasing transmission risk related mortality**: In the base case, the mortality rate used for those without advanced liver disease reflects that of the general population. However, evidence suggests that the mortality rate may be higher due to the participation in transmission activities such as drug abuse. In this scenario, for the younger cohort, the model assumed 80% were IDU with a hazard ratio of 9.1 applied on the baseline mortality rate [1, 2].
4. **Patent expiration of HCV drugs**: Current DAAs were approved for use by Health Canada in 2014. Given that many drugs go off patent within 10 years of market access, a scenario was considered where treatment costs were reduced by 66% in 2024 based on a systematic review of the impact of patent expiry on drug prices [3]. In this scenario, a delayed screening comparator was considered.
5. **Cancer screening agency model of care**: The base case analysis assumed that increased awareness of birth cohort screening, perhaps perpetuated by federal agencies and advocacy groups, would lead to more people coming forward for testing as well as clinicians screening more indiscriminately. This was observed in the US with CDC birth cohort screening recommendation and had been how screening was evaluated in the past. In this scenario analysis, we assume that a provincial agency exclusively for HCV screening is established, akin to that seen in cancer screening. Such an agency would have operating costs shown in Table S2.1 and would increase screening rates in all birth cohorts by another 16% based on data from colorectal cancer screening [4].
6. **Decreasing diagnostic yield:** The base case analysis assumed that for the one-time birth cohort screening intervention, screening rate in the birth cohort will be increased by 50% in the years after the recommendation and we also assumed this rate increase remained constant over time and applied equally to those with or without HCV. In this scenario analysis, we assume that the rate increase for those without HCV is 2 – 4 times higher than those with HCV.
7. **Discount rate:** The base case analysis used a 1.5% discount rate based on the Canadian economic evaluation guideline. In this scenario analysis, we assume a 3% discount rate.

#####

##### Table S2.1: Anticipated operating costs of a provincial agency for HCV screening

| Item | Unit cost | Total cost |
| --- | --- | --- |
| Fixed costs | | |
| Provincial screening lead (1 FTE) | $125,000 (+24% benefits) | $155,000 per year |
| Clinical lead (0.2 FTE) | $250,000 per year | $50,000 per year |
| Non clinical staff (10 FTE) | $50,000 per year (+24% benefits) | $620,000 per year |
| Computers, software & supplies | $5,000 per year | $5,000 per year |
| Operating space | $200,000 | $200,000 per year |
|  | TOTAL fixed costs | $1.03 million per year |
| Variable costs | | |
| Cost of invitations (500,000 sent per year) | $2 | $1 million per year |

## S2.2 Threshold analysis

## The prevalence of the disease usually considered as a critical parameter for one time screening intervention, so we conducted a threshold analysis to explore what the minimum prevalence would have to be for the one time screening intervention to be considered cost-effective under the $50,000/QALY willingness-to-pay threshold.

## S2.3 Results of scenario analyses

### Scenario 1: Improving linkage to care

The model was re-run assuming that there was no loss to follow-up between a positive antibody test and an RNA test.

##### Table S2.2: Results of scenario analysis 1

| Screening birth cohort (1964 onwards) | | | | | | | |
| --- | --- | --- | --- | --- | --- | --- | --- |
|  | **Cost (risk-based screening(status quo))** | **Cost (one-time birth cohort screening)** | **∆ (cost)** | **QALYs (risk-based screening(status quo))** | **QALYs (one-time birth cohort screening)** | **∆ (QALYs)** | **ICER** |
| AB | $321,031 | $321,113 | $81.66 | 25.3518 | 25.3537 | 0.0019 | $42,103 |
| BC | $210,790 | $210,869 | $79.03 | 25.3467 | 25.3495 | 0.0028 | $28,111 |
| MB | $278,385 | $278,459 | $74.77 | 25.3518 | 25.3537 | 0.0019 | $38,548 |
| NB | $229,716 | $229,799 | $82.92 | 25.3453 | 25.3481 | 0.0028 | $29,578 |
| NL | $303,393 | $303,487 | $94.08 | 25.3453 | 25.3481 | 0.0028 | $33,557 |
| NS | $247,126 | $247,212 | $85.83 | 25.3453 | 25.3481 | 0.0028 | $30,614 |
| ON | $221,862 | $221,942 | $79.69 | 25.3477 | 25.3505 | 0.0028 | $28,633 |
| PE | $256,275 | $256,358 | $82.88 | 25.3453 | 25.3481 | 0.0028 | $29,561 |
| QC | $223,258 | $223,341 | $82.75 | 25.3448 | 25.3479 | 0.0031 | $26,399 |
| SK | $267,864 | $267,941 | $76.17 | 25.3518 | 25.3537 | 0.0019 | $39,275 |
| Screening birth cohort (1945-64) | | | | | | | |
|  | **Cost (risk-based screening(status quo))** | **Cost (one-time birth cohort screening)** | **∆ (cost)** | **QALYs (risk-based screening(status quo))** | **QALYs (one-time birth cohort screening)** | **∆ (QALYs)** | **ICER** |
| AB | $347,020 | $347,126 | $105.99 | 15.6434 | 15.6458 | 0.0024 | $44,254 |
| BC | $216,628 | $216,689 | $61.10 | 15.6455 | 15.6474 | 0.0019 | $32,623 |
| MB | $301,468 | $301,562 | $94.28 | 15.6434 | 15.6458 | 0.0024 | $39,362 |
| NB | $249,409 | $249,491 | $81.83 | 15.6417 | 15.6440 | 0.0022 | $36,996 |
| NL | $336,520 | $336,621 | $100.95 | 15.6417 | 15.6440 | 0.0022 | $45,645 |
| NS | $269,505 | $269,592 | $87.53 | 15.6417 | 15.6440 | 0.0022 | $39,577 |
| ON | $239,907 | $239,969 | $62.70 | 15.6497 | 15.6514 | 0.0017 | $37,115 |
| PE | $293,329 | $293,427 | $98.11 | 15.6417 | 15.6440 | 0.0022 | $44,357 |
| QC | $247,525 | $247,601 | $76.07 | 15.6513 | 15.6534 | 0.0022 | $34,988 |
| SK | $281,394 | $281,487 | $92.87 | 15.6434 | 15.6458 | 0.0024 | $38,773 |
| Screening birth cohort (before 1945) | | | | | | | |
|  | **Cost (risk-based screening(status quo))** | **Cost (one-time birth cohort screening)** | **∆ (cost)** | **QALYs (risk-based screening(status quo))** | **QALYs (one-time birth cohort screening)** | **∆ (QALYs)** | **ICER** |
| AB | $343,298 | $343,338 | $40.84 | 6.1770 | 6.1772 | 0.0002 | $173,794 |
| BC | $188,203 | $188,218 | $15.38 | 6.1785 | 6.1786 | 0.0001 | $136,257 |
| MB | $297,845 | $297,883 | $37.70 | 6.1770 | 6.1772 | 0.0002 | $160,436 |
| NB | $232,557 | $232,586 | $29.04 | 6.1768 | 6.1770 | 0.0002 | $148,367 |
| NL | $346,199 | $346,233 | $33.88 | 6.1768 | 6.1770 | 0.0002 | $173,087 |
| NS | $267,043 | $267,073 | $30.58 | 6.1768 | 6.1770 | 0.0002 | $156,244 |
| ON | $222,449 | $222,461 | $11.75 | 6.1786 | 6.1787 | 0.0001 | $136,302 |
| PE | $325,013 | $325,046 | $33.57 | 6.1768 | 6.1770 | 0.0002 | $171,531 |
| QC | $243,244 | $243,257 | $13.48 | 6.1785 | 6.1786 | 0.0001 | $148,090 |
| SK | $269,985 | $270,023 | $37.56 | 6.1770 | 6.1772 | 0.0002 | $159,838 |

Abbreviations: AB, Alberta; BC, British Columbia; MB, Manitoba; NB, New Brunswick; NL, Newfoundland & Labrador; NS, Nova Scotia; ON, Ontario; PE, Prince Edward Island; QC, Quebec; SK, Saskatchewan ; δ, difference; QALY, Quality adjusted Life Years; ICER, Incremental Cost Effectiveness Ratio.

Improving linkage to care increased the cost-effectiveness of screening in all birth cohorts. However, the improvement was slight and the ICER did not change the conclusions about cost-effectiveness using a $50,000 per QALY threshold. The older the cohort the larger the impact of improved linkage to care because the potential to benefit from a HCV cure would be severely reduced the longer they had to wait. For the younger cohort they had more time to be re-tested before advanced liver disease occurred.

### Scenario 2: Reducing unnecessary re-testing

The model was re-run assuming that those with a negative antibody test would not be re-tested during the duration of the model simulation.

##### Table S2.3: Results of scenario analysis 2

| Screening birth cohort (1964 onwards) | | | | | | | |
| --- | --- | --- | --- | --- | --- | --- | --- |
|  | **Cost (risk-based screening(status quo))** | **Cost (one-time birth cohort screening)** | **∆ (cost)** | **QALYs (risk-based screening(status quo))** | **QALYs (one-time birth cohort screening)** | **∆ (QALYs)** | **ICER** |
| AB | $321,031 | $321,073 | $41.68 | 25.3518 | 25.3530 | 0.0013 | $32,687 |
| BC | $210,790 | $210,830 | $39.78 | 25.3467 | 25.3485 | 0.0018 | $22,276 |
| MB | $278,385 | $278,422 | $37.37 | 25.3518 | 25.3530 | 0.0013 | $29,307 |
| NB | $229,716 | $229,759 | $43.02 | 25.3453 | 25.3471 | 0.0018 | $23,530 |
| NL | $303,393 | $303,443 | $50.17 | 25.3453 | 25.3471 | 0.0018 | $27,441 |
| NS | $247,126 | $247,171 | $44.85 | 25.3453 | 25.3471 | 0.0018 | $24,530 |
| ON | $221,862 | $221,903 | $40.37 | 25.3477 | 25.3495 | 0.0018 | $22,761 |
| PE | $256,275 | $256,318 | $43.26 | 25.3453 | 25.3471 | 0.0018 | $23,660 |
| QC | $223,258 | $223,300 | $42.43 | 25.3448 | 25.3468 | 0.0020 | $21,334 |
| SK | $267,864 | $267,903 | $38.11 | 25.3518 | 25.3530 | 0.0013 | $29,881 |
| Screening birth cohort (1945-64) | | | | | | | |
|  | **Cost (risk-based screening(status quo))** | **Cost (one-time birth cohort screening)** | **∆ (cost)** | **QALYs (risk-based screening(status quo))** | **QALYs (one-time birth cohort screening)** | **∆ (QALYs)** | **ICER** |
| AB | $347,020 | $347,072 | $52.61 | 15.6434 | 15.6447 | 0.0013 | $39,260 |
| BC | $216,628 | $216,655 | $27.11 | 15.6455 | 15.6466 | 0.0011 | $25,730 |
| MB | $301,468 | $301,513 | $45.90 | 15.6434 | 15.6447 | 0.0013 | $34,256 |
| NB | $249,409 | $249,448 | $38.72 | 15.6417 | 15.6430 | 0.0012 | $31,283 |
| NL | $336,520 | $336,569 | $49.67 | 15.6417 | 15.6430 | 0.0012 | $40,125 |
| NS | $269,505 | $269,547 | $41.99 | 15.6417 | 15.6430 | 0.0012 | $33,926 |
| ON | $239,907 | $239,934 | $27.58 | 15.6497 | 15.6507 | 0.0009 | $29,206 |
| PE | $293,329 | $293,377 | $48.06 | 15.6417 | 15.6430 | 0.0012 | $38,829 |
| QC | $247,525 | $247,561 | $35.61 | 15.6513 | 15.6525 | 0.0012 | $29,202 |
| SK | $281,394 | $281,439 | $45.13 | 15.6434 | 15.6447 | 0.0013 | $33,682 |
| Screening birth cohort (before 1945) | | | | | | | |
|  | **Cost (risk-based screening(status quo))** | **Cost (one-time birth cohort screening)** | **∆ (cost)** | **QALYs (risk-based screening(status quo))** | **QALYs (one-time birth cohort screening)** | **∆ (QALYs)** | **ICER** |
| AB | $343,298 | $343,318 | $20.43 | 6.1770 | 6.1771 | 0.0001 | $167,695 |
| BC | $188,203 | $188,210 | $7.03 | 6.1785 | 6.1786 | 0.0001 | $120,203 |
| MB | $297,845 | $297,864 | $18.79 | 6.1770 | 6.1771 | 0.0001 | $154,229 |
| NB | $232,557 | $232,571 | $14.21 | 6.1768 | 6.1769 | 0.0001 | $140,272 |
| NL | $346,199 | $346,216 | $16.74 | 6.1768 | 6.1769 | 0.0001 | $165,182 |
| NS | $267,043 | $267,058 | $15.02 | 6.1768 | 6.1769 | 0.0001 | $148,210 |
| ON | $222,449 | $222,454 | $5.11 | 6.1786 | 6.1787 | 0.0000 | $114,418 |
| PE | $325,013 | $325,029 | $16.58 | 6.1768 | 6.1769 | 0.0001 | $163,628 |
| QC | $243,244 | $243,250 | $6.03 | 6.1785 | 6.1786 | 0.0000 | $127,745 |
| SK | $269,985 | $270,004 | $18.73 | 6.1770 | 6.1771 | 0.0001 | $153,687 |

Abbreviations: AB, Alberta; BC, British Columbia; MB, Manitoba; NB, New Brunswick; NL, Newfoundland & Labrador; NS, Nova Scotia; ON, Ontario; PE, Prince Edward Island; QC, Quebec; SK, Saskatchewan ; δ, difference; QALY, Quality adjusted Life Years; ICER, Incremental Cost Effectiveness Ratio.

Reducing the number of ‘unnecessary’ antibody tests reduced the incremental cost of additional screening and left the incremental QALYs unchanged. As the reduction in incremental costs was only slight, the overall change to the ICER across birth cohorts remained largely unchanged. The younger the cohort the larger the impact on incremental costs as these individuals had more opportunities to be re-tested.

### Scenario 3: Increasing transmission risk related mortality

In this scenario, for the younger cohort, the model assumed 80% were IDU with a hazard ratio of 9.1 applied to the baseline mortality rate [1, 2].

##### Table S2.4: Results of scenario analysis 3

| Screening birth cohort (1964 onwards) | | | | | | | |
| --- | --- | --- | --- | --- | --- | --- | --- |
|  | **Cost (risk-based screening(status quo))** | **Cost (one-time birth cohort screening)** | **∆ (cost)** | **QALYs (risk-based screening(status quo))** | **QALYs (one-time birth cohort screening)** | **∆ (QALYs)** | **ICER** |
| AB | $318,699 | $318,752 | $52.73 | 25.2543 | 25.2548 | 0.0004 | $117,780 |
| BC | $209,256 | $209,304 | $48.05 | 25.2429 | 25.2435 | 0.0006 | $74,191 |
| MB | $276,408 | $276,458 | $49.78 | 25.2543 | 25.2548 | 0.0004 | $111,208 |
| NB | $227,484 | $227,539 | $54.69 | 25.2157 | 25.2164 | 0.0006 | $84,977 |
| NL | $300,359 | $300,417 | $58.30 | 25.2157 | 25.2164 | 0.0006 | $90,579 |
| NS | $244,686 | $244,741 | $55.52 | 25.2157 | 25.2164 | 0.0006 | $86,261 |
| ON | $220,148 | $220,196 | $47.92 | 25.2458 | 25.2464 | 0.0006 | $74,509 |
| PE | $253,486 | $253,538 | $52.64 | 25.2157 | 25.2164 | 0.0006 | $81,793 |
| QC | $221,326 | $221,374 | $48.30 | 25.2327 | 25.2334 | 0.0007 | $66,882 |
| SK | $265,970 | $266,020 | $50.50 | 25.2543 | 25.2548 | 0.0004 | $112,800 |

Abbreviations: AB, Alberta; BC, British Columbia; MB, Manitoba; NB, New Brunswick; NL, Newfoundland & Labrador; NS, Nova Scotia; ON, Ontario; PE, Prince Edward Island; QC, Quebec; SK, Saskatchewan ; δ, difference; QALY, Quality adjusted Life Years; ICER, Incremental Cost Effectiveness Ratio.

For birth cohort after 1964, increasing the mortality risk in HCV positive people decreased the incremental cost of screening but also decreased the incremental benefit. The overall change to the ICER was significant, causing screening to become not cost-effective at a $50,000 per QALY threshold.

### Scenario 4: Patent expiration of HCV drugs

In this scenario, the cost of HCV treatment dropped to 66% of $46,000 after the model had run for 5 years and a new delayed screening strategy was adopted.

##### Table S2.5: Results of scenario analysis 4

| Screening birth cohort (1964 onwards) | | | | | | | |
| --- | --- | --- | --- | --- | --- | --- | --- |
|  | **Strategies** | **Cost** | **∆ cost** | **QALYs** | **∆ (QALYs)** | **ICER** | **Dominance** |
| AB | Risk-based screening (status quo) | $320,973 |  | 25.3518 |  |  | undominated |
|  | Delayed one-time birth cohort screening | $321,013 | $39.50 | 25.3529 | 0.0011 | $36,244 | undominated |
|  | One-time birth cohort screening | $321,025 | $51.43 | 25.3531 | 0.0013 | $40,298 | undominated |
| BC | Risk-based screening (status quo) | $210,731 |  | 25.3467 |  |  | undominated |
|  | Delayed one-time birth cohort screening | $210,768 | $36.22 | 25.3481 | 0.0013 | $27,412 | undominated |
|  | One-time birth cohort screening | $210,781 | $49.43 | 25.3485 | 0.0018 | $27,661 | undominated |
| MB | Risk-based screening (status quo) | $278,327 |  | 25.3518 |  |  | undominated |
|  | Delayed one-time birth cohort screening | $278,363 | $36.03 | 25.3529 | 0.0011 | $33,052 | undominated |
|  | One-time birth cohort screening | $278,374 | $47.12 | 25.3531 | 0.0013 | $36,918 | undominated |
| NB | Risk-based screening (status quo) | $229,649 |  | 25.3453 |  |  | undominated |
|  | Delayed one-time birth cohort screening | $229,686 | $37.72 | 25.3467 | 0.0015 | $25,971 | undominated |
|  | One-time birth cohort screening | $229,702 | $53.58 | 25.3471 | 0.0018 | $29,281 | undominated |
| NL | Risk-based screening (status quo) | $303,325 |  | 25.3453 |  |  | undominated |
|  | Delayed one-time birth cohort screening | $303,369 | $43.27 | 25.3467 | 0.0015 | $29,797 | undominated |
|  | One-time birth cohort screening | $303,386 | $60.74 | 25.3471 | 0.0018 | $33,193 | undominated |
| NS | Risk-based screening (status quo) | $247,059 |  | 25.3453 |  |  | undominated |
|  | Delayed one-time birth cohort screening | $247,098 | $39.09 | 25.3467 | 0.0015 | $26,918 | undominated |
|  | One-time birth cohort screening | $247,114 | $55.41 | 25.3471 | 0.0018 | $30,281 | undominated |
| ON | Risk-based screening (status quo) | $221,803 |  | 25.3477 |  |  | undominated |
|  | Delayed one-time birth cohort screening | $221,839 | $36.16 | 25.3490 | 0.0013 | $27,271 | undominated |
|  | One-time birth cohort screening | $221,853 | $50.14 | 25.3495 | 0.0018 | $28,248 | undominated |
| PE | Risk-based screening (status quo) | $256,207 |  | 25.3453 |  |  | undominated |
|  | Delayed one-time birth cohort screening | $256,245 | $37.88 | 25.3467 | 0.0015 | $26,085 | undominated |
|  | One-time birth cohort screening | $256,261 | $53.81 | 25.3471 | 0.0018 | $29,408 | undominated |
| QC | Risk-based screening (status quo) | $223,196 |  | 25.3448 |  |  | undominated |
|  | Delayed one-time birth cohort screening | $223,233 | $36.95 | 25.3463 | 0.0014 | $25,556 | undominated |
|  | One-time birth cohort screening | $223,248 | $52.33 | 25.3468 | 0.0020 | $26,292 | undominated |
| SK | Risk-based screening (status quo) | $267,807 |  | 25.3518 |  |  | undominated |
|  | Delayed one-time birth cohort screening | $267,843 | $36.34 | 25.3529 | 0.0011 | $33,340 | undominated |
|  | One-time birth cohort screening | $267,855 | $47.85 | 25.3531 | 0.0013 | $37,491 | undominated |
| Screening birth cohort (1945-64) | | | | | | | |
|  | **Strategies** | **Cost** | **∆ cost** | **QALYs** | **∆ (QALYs)** | **ICER** | **Dominance** |
| AB | Risk-based screening (status quo) | $347,004 |  | 15.6434 |  |  | undominated |
|  | Delayed one-time birth cohort screening | $347,034 | $30.42 | 15.6440 | 0.0006 | $52,647 | ext. dominated |
|  | One-time birth cohort screening | $347,064 | $60.56 | 15.6447 | 0.0013 | $45,199 | undominated |
| BC | Risk-based screening (status quo) | $216,614 |  | 15.6455 |  |  | undominated |
|  | Delayed one-time birth cohort screening | $216,632 | $18.27 | 15.6460 | 0.0005 | $38,128 | ext. dominated |
|  | One-time birth cohort screening | $216,649 | $35.31 | 15.6466 | 0.0011 | $33,515 | undominated |
| MB | Risk-based screening (status quo) | $301,452 |  | 15.6434 |  |  | undominated |
|  | Delayed one-time birth cohort screening | $301,479 | $26.93 | 15.6440 | 0.0006 | $46,616 | ext. dominated |
|  | One-time birth cohort screening | $301,506 | $53.86 | 15.6447 | 0.0013 | $40,194 | undominated |
| NB | Risk-based screening (status quo) | $249,394 |  | 15.6417 |  |  | undominated |
|  | Delayed one-time birth cohort screening | $249,417 | $23.18 | 15.6423 | 0.0005 | $43,308 | ext. dominated |
|  | One-time birth cohort screening | $249,441 | $46.80 | 15.6430 | 0.0012 | $37,809 | undominated |
| NL | Risk-based screening (status quo) | $336,505 |  | 15.6417 |  |  | undominated |
|  | Delayed one-time birth cohort screening | $336,534 | $28.84 | 15.6423 | 0.0005 | $53,886 | ext. dominated |
|  | One-time birth cohort screening | $336,562 | $57.74 | 15.6430 | 0.0012 | $46,652 | undominated |
| NS | Risk-based screening (status quo) | $269,490 |  | 15.6417 |  |  | undominated |
|  | Delayed one-time birth cohort screening | $269,515 | $24.91 | 15.6423 | 0.0005 | $46,529 | ext. dominated |
|  | One-time birth cohort screening | $269,540 | $50.07 | 15.6430 | 0.0012 | $40,453 | undominated |
| ON | Risk-based screening (status quo) | $239,896 |  | 15.6497 |  |  | undominated |
|  | Delayed one-time birth cohort screening | $239,914 | $17.99 | 15.6502 | 0.0004 | $44,631 | ext. dominated |
|  | One-time birth cohort screening | $239,932 | $36.17 | 15.6507 | 0.0009 | $38,309 | undominated |
| PE | Risk-based screening (status quo) | $293,314 |  | 15.6417 |  |  | undominated |
|  | Delayed one-time birth cohort screening | $293,342 | $28.12 | 15.6423 | 0.0005 | $52,543 | ext. dominated |
|  | One-time birth cohort screening | $293,370 | $56.14 | 15.6430 | 0.0012 | $45,356 | undominated |
| QC | Risk-based screening (status quo) | $247,510 |  | 15.6513 |  |  | undominated |
|  | Delayed one-time birth cohort screening | $247,532 | $22.28 | 15.6518 | 0.0005 | $41,370 | ext. dominated |
|  | One-time birth cohort screening | $247,553 | $43.67 | 15.6525 | 0.0012 | $35,817 | undominated |
| SK | Risk-based screening (status quo) | $281,378 |  | 15.6434 |  |  | undominated |
|  | Delayed one-time birth cohort screening | $281,405 | $26.69 | 15.6440 | 0.0006 | $46,192 | ext. dominated |
|  | One-time birth cohort screening | $281,432 | $53.09 | 15.6447 | 0.0013 | $39,621 | undominated |
| Screening birth cohort (before 1945) | | | | | | | |
|  | **Strategies** | **Cost** | **∆ cost** | **QALYs** | **∆ (QALYs)** | **ICER** | **Dominance** |
| AB | Risk-based screening (status quo) | $343,293 |  | 6.1770 |  |  | undominated |
|  | Delayed one-time birth cohort screening | $343,299 | $6.47 | 6.1770 | 0.0000 | $209,125 | ext. dominated |
|  | One-time birth cohort screening | $343,313 | $20.19 | 6.1771 | 0.0001 | $165,710 | undominated |
| BC | Risk-based screening (status quo) | $188,201 |  | 6.1785 |  |  | undominated |
|  | Delayed one-time birth cohort screening | $188,203 | $2.49 | 6.1785 | 0.0000 | $170,027 | ext. dominated |
|  | One-time birth cohort screening | $188,208 | $7.61 | 6.1786 | 0.0001 | $130,127 | undominated |
| MB | Risk-based screening (status quo) | $297,841 |  | 6.1770 |  |  | undominated |
|  | Delayed one-time birth cohort screening | $297,847 | $5.93 | 6.1770 | 0.0000 | $191,824 | ext. dominated |
|  | One-time birth cohort screening | $297,859 | $18.55 | 6.1771 | 0.0001 | $152,244 | undominated |
| NB | Risk-based screening (status quo) | $232,554 |  | 6.1768 |  |  | undominated |
|  | Delayed one-time birth cohort screening | $232,558 | $4.43 | 6.1768 | 0.0000 | $183,545 | ext. dominated |
|  | One-time birth cohort screening | $232,568 | $14.32 | 6.1769 | 0.0001 | $141,290 | undominated |
| NL | Risk-based screening (status quo) | $346,195 |  | 6.1768 |  |  | undominated |
|  | Delayed one-time birth cohort screening | $346,201 | $5.21 | 6.1768 | 0.0000 | $215,631 | ext. dominated |
|  | One-time birth cohort screening | $346,212 | $16.84 | 6.1769 | 0.0001 | $166,199 | undominated |
| NS | Risk-based screening (status quo) | $267,039 |  | 6.1768 |  |  | undominated |
|  | Delayed one-time birth cohort screening | $267,044 | $4.68 | 6.1768 | 0.0000 | $193,795 | ext. dominated |
|  | One-time birth cohort screening | $267,054 | $15.12 | 6.1769 | 0.0001 | $149,228 | undominated |
| ON | Risk-based screening (status quo) | $222,448 |  | 6.1786 |  |  | undominated |
|  | Delayed one-time birth cohort screening | $222,450 | $1.89 | 6.1786 | 0.0000 | $178,408 | ext. dominated |
|  | One-time birth cohort screening | $222,454 | $5.90 | 6.1787 | 0.0000 | $132,177 | undominated |
| PE | Risk-based screening (status quo) | $325,009 |  | 6.1768 |  |  | undominated |
|  | Delayed one-time birth cohort screening | $325,014 | $5.17 | 6.1768 | 0.0000 | $214,159 | ext. dominated |
|  | One-time birth cohort screening | $325,026 | $16.68 | 6.1769 | 0.0001 | $164,645 | undominated |
| QC | Risk-based screening (status quo) | $243,242 |  | 6.1785 |  |  | undominated |
|  | Delayed one-time birth cohort screening | $243,244 | $2.20 | 6.1786 | 0.0000 | $188,555 | ext. dominated |
|  | One-time birth cohort screening | $243,249 | $6.74 | 6.1786 | 0.0000 | $142,899 | undominated |
| SK | Risk-based screening (status quo) | $269,981 |  | 6.1770 |  |  | undominated |
|  | Delayed one-time birth cohort screening | $269,987 | $5.99 | 6.1770 | 0.0000 | $193,719 | ext. dominated |
|  | One-time birth cohort screening | $269,999 | $18.49 | 6.1771 | 0.0001 | $151,702 | undominated |

Abbreviations: AB, Alberta; BC, British Columbia; MB, Manitoba; NB, New Brunswick; NL, Newfoundland & Labrador; NS, Nova Scotia; ON, Ontario; PE, Prince Edward Island; QC, Quebec; SK, Saskatchewan ; δ, difference; QALY, Quality adjusted Life Years; ICER, Incremental Cost Effectiveness Ratio.

The delayed screening strategy was cost-effective relative to no screening. By screening straight away while drug prices were high, there was a higher incremental cost; however the incremental benefit justified this additional cost. In the younger birth cohort (after 1964) and the baby boomer cohort (1945-64), the ICERs of delayed screening and up-front screening were below the $50,000 per QALY threshold. In the older age cohort, delayed screening was more cost-effective than up front screening but the ICER remained above $50,000 per QALY.

### Scenario 5: Cancer screening agency model of care

It was assumed that a HCV screening agency was set up in Ontario at a cost of $2.03 million a year. Based on evidence from colorectal cancer screening [4], a further 16% screening uptake (i.e. relative risk of 1.73) was assumed.

##### Table S2.6: Results of scenario analysis 5

| Screening birth cohort (1964 onwards) | | | | | | | |
| --- | --- | --- | --- | --- | --- | --- | --- |
|  | **Cost (risk-based screening(status quo))** | **Cost (one-time birth cohort screening)** | **∆ (cost)** | **QALYs (risk-based screening(status quo))** | **QALYs (one-time birth cohort screening)** | **∆ (QALYs)** | **ICER** |
| AB | $321,031 | $321,108 | $76.52 | 25.3518 | 25.3534 | 0.0016 | $46,452 |
| BC | $210,790 | $210,864 | $74.20 | 25.3467 | 25.3491 | 0.0023 | $31,608 |
| MB | $278,385 | $278,456 | $70.81 | 25.3518 | 25.3534 | 0.0016 | $42,989 |
| NB | $229,716 | $229,794 | $77.92 | 25.3453 | 25.3476 | 0.0024 | $32,858 |
| NL | $303,393 | $303,480 | $87.26 | 25.3453 | 25.3476 | 0.0024 | $36,796 |
| NS | $247,126 | $247,207 | $80.33 | 25.3453 | 25.3476 | 0.0024 | $33,875 |
| ON | $221,862 | $221,937 | $74.86 | 25.3477 | 25.3500 | 0.0023 | $32,159 |
| PE | $256,275 | $256,353 | $78.04 | 25.3453 | 25.3476 | 0.0024 | $32,908 |
| QC | $223,258 | $223,335 | $77.48 | 25.3448 | 25.3474 | 0.0026 | $29,619 |
| SK | $267,864 | $267,936 | $71.88 | 25.3518 | 25.3534 | 0.0016 | $43,639 |
| Screening birth cohort (1945-64) | | | | | | | |
|  | **Cost (risk-based screening(status quo))** | **Cost (one-time birth cohort screening)** | **∆ (cost)** | **QALYs (risk-based screening(status quo))** | **QALYs (one-time birth cohort screening)** | **∆ (QALYs)** | **ICER** |
| AB | $347,020 | $347,110 | $90.45 | 15.6434 | 15.6453 | 0.0019 | $48,072 |
| BC | $216,628 | $216,683 | $54.91 | 15.6455 | 15.6470 | 0.0015 | $37,215 |
| MB | $301,468 | $301,549 | $81.14 | 15.6434 | 15.6453 | 0.0019 | $43,124 |
| NB | $249,409 | $249,480 | $71.20 | 15.6417 | 15.6435 | 0.0017 | $40,972 |
| NL | $336,520 | $336,606 | $86.40 | 15.6417 | 15.6435 | 0.0017 | $49,716 |
| NS | $269,505 | $269,581 | $75.74 | 15.6417 | 15.6435 | 0.0017 | $43,583 |
| ON | $239,907 | $239,963 | $55.88 | 15.6497 | 15.6511 | 0.0013 | $42,126 |
| PE | $293,329 | $293,413 | $84.15 | 15.6417 | 15.6435 | 0.0017 | $48,424 |
| QC | $247,525 | $247,592 | $66.78 | 15.6513 | 15.6530 | 0.0017 | $39,052 |
| SK | $281,394 | $281,474 | $80.05 | 15.6434 | 15.6453 | 0.0019 | $42,543 |
| Screening birth cohort (before 1945) | | | | | | | |
|  | **Cost (risk-based screening(status quo))** | **Cost (one-time birth cohort screening)** | **∆ (cost)** | **QALYs (risk-based screening(status quo))** | **QALYs (one-time birth cohort screening)** | **∆ (QALYs)** | **ICER** |
| AB | $343,298 | $343,331 | $33.09 | 6.17700 | 6.17718 | 0.00018 | $186,195 |
| BC | $188,203 | $188,216 | $13.68 | 6.17849 | 6.17858 | 0.00009 | $160,292 |
| MB | $297,845 | $297,876 | $30.71 | 6.17700 | 6.17718 | 0.00018 | $172,782 |
| NB | $232,557 | $232,581 | $24.08 | 6.17676 | 6.17691 | 0.00015 | $162,829 |
| NL | $346,199 | $346,227 | $27.75 | 6.17676 | 6.17691 | 0.00015 | $187,645 |
| NS | $267,043 | $267,068 | $25.25 | 6.17676 | 6.17691 | 0.00015 | $170,736 |
| ON | $222,449 | $222,460 | $10.90 | 6.17861 | 6.17867 | 0.00007 | $167,300 |
| PE | $325,013 | $325,040 | $27.52 | 6.17676 | 6.17691 | 0.00015 | $186,090 |
| QC | $243,244 | $243,256 | $12.22 | 6.17854 | 6.17861 | 0.00007 | $177,618 |
| SK | $269,985 | $270,016 | $30.61 | 6.17700 | 6.17718 | 0.00018 | $172,213 |

Abbreviations: AB, Alberta; BC, British Columbia; MB, Manitoba; NB, New Brunswick; NL, Newfoundland & Labrador; NS, Nova Scotia; ON, Ontario; PE, Prince Edward Island; QC, Quebec; SK, Saskatchewan ; δ, difference; QALY, Quality adjusted Life Years; ICER, Incremental Cost Effectiveness Ratio.

In this hypothetical scenario, the screening agency increased screening rate at an extra 16% compared to the base case. The incremental cost was higher as the expense of the screening agency was considerable. However, this scenario analysis showed that it was still cost-effective with these additional costs accounted for. This shows there is scope to expand screening program.

**Scenario 6: Decreasing diagnostic yield**

The base case analysis assumed that for the one-time birth cohort screening intervention, screening rate in the birth cohort will be increased by 50% in the years after the recommendation and we also assumed this rate increase remained constant over time and applied equally to those with or without HCV. In this scenario analysis, we assume that the rate increase for those without HCV is 2 – 4 times higher than those with HCV.

Table S2.7: Results of scenario analysis 6

| Diagnostic yield decrease by 2 times | | | | | | | |
| --- | --- | --- | --- | --- | --- | --- | --- |
| Screening birth cohort (1964 onwards) | | | | | | | |
|  | **Cost (risk-based screening(status quo))** | **Cost (one-time birth cohort screening)** | **∆ (cost)** | **QALYs (risk-based screening(status quo))** | **QALYs (one-time birth cohort screening)** | **∆ (QALYs)** | **ICER** |
| AB | $321,031 | $321,097 | $65.92 | 25.3518 | 25.3530 | 0.0013 | $51,694 |
| BC | $210,790 | $210,854 | $64.01 | 25.3467 | 25.3485 | 0.0018 | $35,843 |
| MB | $278,385 | $278,446 | $61.61 | 25.3518 | 25.3530 | 0.0013 | $48,315 |
| NB | $229,716 | $229,783 | $67.22 | 25.3453 | 25.3471 | 0.0018 | $36,764 |
| NL | $303,393 | $303,467 | $74.37 | 25.3453 | 25.3471 | 0.0018 | $40,676 |
| NS | $247,126 | $247,195 | $69.05 | 25.3453 | 25.3471 | 0.0018 | $37,765 |
| ON | $221,862 | $221,927 | $64.61 | 25.3477 | 25.3495 | 0.0018 | $36,421 |
| PE | $256,275 | $256,342 | $67.46 | 25.3453 | 25.3471 | 0.0018 | $36,894 |
| QC | $223,258 | $223,324 | $66.65 | 25.3448 | 25.3468 | 0.0020 | $33,510 |
| SK | $267,864 | $267,927 | $62.35 | 25.3518 | 25.3530 | 0.0013 | $48,889 |
| Screening birth cohort (1945-64) | | | | | | | |
|  | **Cost (risk-based screening(status quo))** | **Cost (one-time birth cohort screening)** | **∆ (cost)** | **QALYs (risk-based screening(status quo))** | **QALYs (one-time birth cohort screening)** | **∆ (QALYs)** | **ICER** |
| AB | $347,020 | $347,092 | $72.60 | 15.6434 | 15.6447 | 0.0013 | $54,186 |
| BC | $216,628 | $216,675 | $47.10 | 15.6455 | 15.6466 | 0.0011 | $44,703 |
| MB | $301,468 | $301,533 | $65.90 | 15.6434 | 15.6447 | 0.0013 | $49,181 |
| NB | $249,409 | $249,468 | $58.70 | 15.6417 | 15.6430 | 0.0012 | $47,426 |
| NL | $336,520 | $336,589 | $69.65 | 15.6417 | 15.6430 | 0.0012 | $56,268 |
| NS | $269,505 | $269,567 | $61.97 | 15.6417 | 15.6430 | 0.0012 | $50,069 |
| ON | $239,907 | $239,954 | $47.61 | 15.6497 | 15.6507 | 0.0009 | $50,426 |
| PE | $293,329 | $293,397 | $68.04 | 15.6417 | 15.6430 | 0.0012 | $54,972 |
| QC | $247,525 | $247,581 | $55.66 | 15.6513 | 15.6525 | 0.0012 | $45,646 |
| SK | $281,394 | $281,459 | $65.13 | 15.6434 | 15.6447 | 0.0013 | $48,608 |
| Screening birth cohort (before 1945) | | | | | | | |
|  | **Cost (risk-based screening(status quo))** | **Cost (one-time birth cohort screening)** | **∆ (cost)** | **QALYs (risk-based screening(status quo))** | **QALYs (one-time birth cohort screening)** | **∆ (QALYs)** | **ICER** |
| AB | $343,298 | $343,321 | $22.99 | 6.1770 | 6.1771 | 0.0001 | $188,695 |
| BC | $188,203 | $188,212 | $9.60 | 6.1785 | 6.1786 | 0.0001 | $164,161 |
| MB | $297,845 | $297,867 | $21.35 | 6.1770 | 6.1771 | 0.0001 | $175,229 |
| NB | $232,557 | $232,574 | $16.77 | 6.1768 | 6.1769 | 0.0001 | $165,455 |
| NL | $346,199 | $346,218 | $19.29 | 6.1768 | 6.1769 | 0.0001 | $190,365 |
| NS | $267,043 | $267,060 | $17.57 | 6.1768 | 6.1769 | 0.0001 | $173,393 |
| ON | $222,449 | $222,457 | $7.68 | 6.1786 | 6.1787 | 0.0000 | $172,023 |
| PE | $325,013 | $325,032 | $19.13 | 6.1768 | 6.1769 | 0.0001 | $188,811 |
| QC | $243,244 | $243,252 | $8.59 | 6.1785 | 6.1786 | 0.0000 | $182,176 |
| SK | $269,985 | $270,006 | $21.29 | 6.1770 | 6.1771 | 0.0001 | $174,687 |

| Diagnostic yield decrease by 3 times | | | | | | | |
| --- | --- | --- | --- | --- | --- | --- | --- |
| Screening birth cohort (1964 onwards) | | | | | | | |
|  | **Cost (risk-based screening(status quo))** | **Cost (one-time birth cohort screening)** | **∆ (cost)** | **QALYs (risk-based screening(status quo))** | **QALYs (one-time birth cohort screening)** | **∆ (QALYs)** | **ICER** |
| AB | $321,031 | $321,109 | $78.04 | 25.3518 | 25.3530 | 0.0013 | $61,198 |
| BC | $210,790 | $210,866 | $76.13 | 25.3467 | 25.3485 | 0.0018 | $42,626 |
| MB | $278,385 | $278,458 | $73.73 | 25.3518 | 25.3530 | 0.0013 | $57,819 |
| NB | $229,716 | $229,795 | $79.32 | 25.3453 | 25.3471 | 0.0018 | $43,381 |
| NL | $303,393 | $303,479 | $86.47 | 25.3453 | 25.3471 | 0.0018 | $47,293 |
| NS | $247,126 | $247,208 | $81.14 | 25.3453 | 25.3471 | 0.0018 | $44,382 |
| ON | $221,862 | $221,939 | $76.72 | 25.3477 | 25.3495 | 0.0018 | $43,252 |
| PE | $256,275 | $256,354 | $79.55 | 25.3453 | 25.3471 | 0.0018 | $43,511 |
| QC | $223,258 | $223,337 | $78.76 | 25.3448 | 25.3468 | 0.0020 | $39,597 |
| SK | $267,864 | $267,939 | $74.47 | 25.3518 | 25.3530 | 0.0013 | $58,393 |
| Screening birth cohort (1945-64) | | | | | | | |
|  | **Cost (risk-based screening(status quo))** | **Cost (one-time birth cohort screening)** | **∆ (cost)** | **QALYs (risk-based screening(status quo))** | **QALYs (one-time birth cohort screening)** | **∆ (QALYs)** | **ICER** |
| AB | $347,020 | $347,102 | $82.60 | 15.6434 | 15.6447 | 0.0013 | $61,649 |
| BC | $216,628 | $216,685 | $57.10 | 15.6455 | 15.6466 | 0.0011 | $54,189 |
| MB | $301,468 | $301,543 | $75.90 | 15.6434 | 15.6447 | 0.0013 | $56,644 |
| NB | $249,409 | $249,478 | $68.69 | 15.6417 | 15.6430 | 0.0012 | $55,497 |
| NL | $336,520 | $336,599 | $79.64 | 15.6417 | 15.6430 | 0.0012 | $64,340 |
| NS | $269,505 | $269,577 | $71.96 | 15.6417 | 15.6430 | 0.0012 | $58,141 |
| ON | $239,907 | $239,964 | $57.63 | 15.6497 | 15.6507 | 0.0009 | $61,035 |
| PE | $293,329 | $293,407 | $78.03 | 15.6417 | 15.6430 | 0.0012 | $63,044 |
| QC | $247,525 | $247,591 | $65.68 | 15.6513 | 15.6525 | 0.0012 | $53,867 |
| SK | $281,394 | $281,469 | $75.13 | 15.6434 | 15.6447 | 0.0013 | $56,071 |
| Screening birth cohort (before 1945) | | | | | | | |
|  | **Cost (risk-based screening(status quo))** | **Cost (one-time birth cohort screening)** | **∆ (cost)** | **QALYs (risk-based screening(status quo))** | **QALYs (one-time birth cohort screening)** | **∆ (QALYs)** | **ICER** |
| AB | $343,298 | $343,322 | $24.27 | 6.1770 | 6.1771 | 0.0001 | $199,196 |
| BC | $188,203 | $188,214 | $10.89 | 6.1785 | 6.1786 | 0.0001 | $186,140 |
| MB | $297,845 | $297,868 | $22.63 | 6.1770 | 6.1771 | 0.0001 | $185,729 |
| NB | $232,557 | $232,575 | $18.04 | 6.1768 | 6.1769 | 0.0001 | $178,046 |
| NL | $346,199 | $346,220 | $20.57 | 6.1768 | 6.1769 | 0.0001 | $202,956 |
| NS | $267,043 | $267,061 | $18.85 | 6.1768 | 6.1769 | 0.0001 | $185,984 |
| ON | $222,449 | $222,458 | $8.97 | 6.1786 | 6.1787 | 0.0000 | $200,825 |
| PE | $325,013 | $325,033 | $20.41 | 6.1768 | 6.1769 | 0.0001 | $201,402 |
| QC | $243,244 | $243,254 | $9.88 | 6.1785 | 6.1786 | 0.0000 | $209,392 |
| SK | $269,985 | $270,008 | $22.57 | 6.1770 | 6.1771 | 0.0001 | $185,187 |

| Diagnostic yield decrease by 4 times | | | | | | | |
| --- | --- | --- | --- | --- | --- | --- | --- |
| Screening birth cohort (1964 onwards) | | | | | | | |
|  | **Cost (risk-based screening(status quo))** | **Cost (one-time birth cohort screening)** | **∆ (cost)** | **QALYs (risk-based screening(status quo))** | **QALYs (one-time birth cohort screening)** | **∆ (QALYs)** | **ICER** |
| AB | $321,031 | $321,121 | $90.16 | 25.3518 | 25.3530 | 0.0013 | $70,702 |
| BC | $210,790 | $210,878 | $88.24 | 25.3467 | 25.3485 | 0.0018 | $49,410 |
| MB | $278,385 | $278,471 | $85.85 | 25.3518 | 25.3530 | 0.0013 | $67,323 |
| NB | $229,716 | $229,807 | $91.41 | 25.3453 | 25.3471 | 0.0018 | $49,998 |
| NL | $303,393 | $303,491 | $98.57 | 25.3453 | 25.3471 | 0.0018 | $53,910 |
| NS | $247,126 | $247,220 | $93.24 | 25.3453 | 25.3471 | 0.0018 | $50,999 |
| ON | $221,862 | $221,951 | $88.84 | 25.3477 | 25.3495 | 0.0018 | $50,082 |
| PE | $256,275 | $256,366 | $91.65 | 25.3453 | 25.3471 | 0.0018 | $50,129 |
| QC | $223,258 | $223,349 | $90.87 | 25.3448 | 25.3468 | 0.0020 | $45,685 |
| SK | $267,864 | $267,951 | $86.58 | 25.3518 | 25.3530 | 0.0013 | $67,897 |
| Screening birth cohort (1945-64) | | | | | | | |
|  | **Cost (risk-based screening(status quo))** | **Cost (one-time birth cohort screening)** | **∆ (cost)** | **QALYs (risk-based screening(status quo))** | **QALYs (one-time birth cohort screening)** | **∆ (QALYs)** | **ICER** |
| AB | $347,020 | $347,112 | $92.60 | 15.6434 | 15.6447 | 0.0013 | $69,111 |
| BC | $216,628 | $216,695 | $67.09 | 15.6455 | 15.6466 | 0.0011 | $63,675 |
| MB | $301,468 | $301,553 | $85.90 | 15.6434 | 15.6447 | 0.0013 | $64,107 |
| NB | $249,409 | $249,488 | $78.68 | 15.6417 | 15.6430 | 0.0012 | $63,569 |
| NL | $336,520 | $336,609 | $89.63 | 15.6417 | 15.6430 | 0.0012 | $72,411 |
| NS | $269,505 | $269,587 | $81.96 | 15.6417 | 15.6430 | 0.0012 | $66,212 |
| ON | $239,907 | $239,974 | $67.65 | 15.6497 | 15.6507 | 0.0009 | $71,645 |
| PE | $293,329 | $293,417 | $88.02 | 15.6417 | 15.6430 | 0.0012 | $71,115 |
| QC | $247,525 | $247,601 | $75.71 | 15.6513 | 15.6525 | 0.0012 | $62,089 |
| SK | $281,394 | $281,479 | $85.13 | 15.6434 | 15.6447 | 0.0013 | $63,533 |
| Screening birth cohort (before 1945) | | | | | | | |
|  | **Cost (risk-based screening(status quo))** | **Cost (one-time birth cohort screening)** | **∆ (cost)** | **QALYs (risk-based screening(status quo))** | **QALYs (one-time birth cohort screening)** | **∆ (QALYs)** | **ICER** |
| AB | $343,298 | $343,323 | $25.55 | 6.1770 | 6.1771 | 0.0001 | $209,696 |
| BC | $188,203 | $188,215 | $12.18 | 6.1785 | 6.1786 | 0.0001 | $208,119 |
| MB | $297,845 | $297,869 | $23.91 | 6.1770 | 6.1771 | 0.0001 | $196,229 |
| NB | $232,557 | $232,576 | $19.32 | 6.1768 | 6.1769 | 0.0001 | $190,637 |
| NL | $346,199 | $346,221 | $21.84 | 6.1768 | 6.1769 | 0.0001 | $215,547 |
| NS | $267,043 | $267,063 | $20.12 | 6.1768 | 6.1769 | 0.0001 | $198,575 |
| ON | $222,449 | $222,459 | $10.25 | 6.1786 | 6.1787 | 0.0000 | $229,628 |
| PE | $325,013 | $325,034 | $21.69 | 6.1768 | 6.1769 | 0.0001 | $213,993 |
| QC | $243,244 | $243,255 | $11.16 | 6.1785 | 6.1786 | 0.0000 | $236,607 |
| SK | $269,985 | $270,009 | $23.85 | 6.1770 | 6.1771 | 0.0001 | $195,687 |

In this hypothetical scenario, if the rate increase for those without HCV is 3 times or higher than those with HCV. The cost-effectiveness conclusion in the base case analysis is likely to change.

**Scenario 7: Discount rate**

The base case analysis used a 1.5% discount rate based on the Canadian economic evaluation guideline. In this scenario analysis, we assume a 3% discount rate. Table S2.8 summarize the result.

Table S2.8: Results of scenario analysis 7

| Screening birth cohort (1964 onwards) | | | | | | | |
| --- | --- | --- | --- | --- | --- | --- | --- |
|  | **Cost (risk-based screening(status quo))** | **Cost (one-time birth cohort screening)** | **∆ (cost)** | **QALYs (risk-based screening(status quo))** | **QALYs (one-time birth cohort screening)** | **∆ (QALYs)** | **ICER** |
| AB | $206,604 | $206,652 | $47.89 | 19.6044 | 19.6053 | 0.0008 | $56,830 |
| BC | $139,700 | $139,746 | $45.27 | 19.6008 | 19.6020 | 0.0012 | $37,982 |
| MB | $178,915 | $178,960 | $44.55 | 19.6044 | 19.6053 | 0.0008 | $52,876 |
| NB | $148,400 | $148,450 | $50.14 | 19.5996 | 19.6008 | 0.0012 | $41,310 |
| NL | $192,528 | $192,583 | $55.25 | 19.5996 | 19.6008 | 0.0012 | $45,519 |
| NS | $158,481 | $158,533 | $51.43 | 19.5996 | 19.6008 | 0.0012 | $42,373 |
| ON | $143,581 | $143,626 | $45.65 | 19.6015 | 19.6027 | 0.0012 | $38,563 |
| PE | $159,262 | $159,312 | $49.61 | 19.5996 | 19.6008 | 0.0012 | $40,870 |
| QC | $142,173 | $142,220 | $47.08 | 19.5994 | 19.6007 | 0.0013 | $35,419 |
| SK | $174,826 | $174,872 | $45.24 | 19.6044 | 19.6053 | 0.0008 | $53,686 |
| Screening birth cohort (1945-64) | | | | | | | |
|  | **Cost (risk-based screening(status quo))** | **Cost (one-time birth cohort screening)** | **∆ (cost)** | **QALYs (risk-based screening(status quo))** | **QALYs (one-time birth cohort screening)** | **∆ (QALYs)** | **ICER** |
| AB | $264,250 | $264,299 | $49.19 | 13.1760 | 13.1770 | 0.0010 | $48,590 |
| BC | $168,139 | $168,171 | $31.28 | 13.1776 | 13.1784 | 0.0008 | $39,421 |
| MB | $229,395 | $229,439 | $44.61 | 13.1760 | 13.1770 | 0.0010 | $44,063 |
| NB | $191,494 | $191,534 | $39.40 | 13.1746 | 13.1755 | 0.0009 | $42,137 |
| NL | $254,692 | $254,739 | $46.85 | 13.1746 | 13.1755 | 0.0009 | $50,112 |
| NS | $205,214 | $205,255 | $41.59 | 13.1746 | 13.1755 | 0.0009 | $44,486 |
| ON | $184,344 | $184,374 | $30.33 | 13.1812 | 13.1820 | 0.0007 | $42,499 |
| PE | $219,364 | $219,410 | $45.60 | 13.1746 | 13.1755 | 0.0009 | $48,775 |
| QC | $188,345 | $188,383 | $37.39 | 13.1826 | 13.1835 | 0.0009 | $40,613 |
| SK | $215,251 | $215,295 | $43.98 | 13.1760 | 13.1770 | 0.0010 | $43,445 |
| Screening birth cohort (before 1945) | | | | | | | |
|  | **Cost (risk-based screening(status quo))** | **Cost (one-time birth cohort screening)** | **∆ (cost)** | **QALYs (risk-based screening(status quo))** | **QALYs (one-time birth cohort screening)** | **∆ (QALYs)** | **ICER** |
| AB | $306,760 | $306,780 | $19.87 | 5.6504 | 5.6505 | 0.0001 | $189,532 |
| BC | $168,777 | $168,785 | $7.68 | 5.6516 | 5.6517 | 0.0001 | $152,543 |
| MB | $265,369 | $265,387 | $18.42 | 5.6504 | 5.6505 | 0.0001 | $175,739 |
| NB | $208,332 | $208,346 | $14.20 | 5.6502 | 5.6503 | 0.0001 | $162,913 |
| NL | $308,983 | $309,000 | $16.40 | 5.6502 | 5.6503 | 0.0001 | $188,188 |
| NS | $238,966 | $238,981 | $14.90 | 5.6502 | 5.6503 | 0.0001 | $170,964 |
| ON | $199,168 | $199,174 | $5.88 | 5.6517 | 5.6518 | 0.0000 | $153,105 |
| PE | $290,819 | $290,835 | $16.26 | 5.6502 | 5.6503 | 0.0001 | $186,559 |
| QC | $217,259 | $217,266 | $6.73 | 5.6517 | 5.6517 | 0.0000 | $166,061 |
| SK | $241,316 | $241,334 | $18.34 | 5.6504 | 5.6505 | 0.0001 | $174,965 |

## S2.4 Results of threshold analysis

## At a willingness-to-pay threshold of $50,000/QALY, the minimum prevalence for each province required for the one time screening intervention to be cost-effective is summarized in table S2.7.

##### Table S2.7: Results of threshold analysis

| Screening birth cohort: after 1964 | | |
| --- | --- | --- |
| Province | Population size | Minimum Prevalence (Threshold) |
| AB | 2,099,424 | 0.039% |
| BC | 2,216,622 | 0.010% |
| MB | 601,875 | 0.010% |
| NB | 322,030 | 0.010% |
| NL | 227,993 | 0.010% |
| NS | 401,427 | 0.010% |
| ON | 6,395,336 | 0.010% |
| PE | 61,470 | 0.010% |
| QC | 3,640,893 | 0.010% |
| SK | 517,454 | 0.010% |
| Screening birth cohort: 1945-1964 | | |
| Province | Population size | Minimum Prevalence (Threshold) |
| AB | 954,962 | 0.966% |
| BC | 1,328,001 | 0.408% |
| MB | 316,516 | 0.517% |
| NB | 233,353 | 0.483% |
| NL | 165,137 | 1.432% |
| NS | 285,935 | 0.602% |
| ON | 3,619,439 | 0.496% |
| PE | 43,202 | 1.112% |
| QC | 2,298,136 | 0.320% |
| SK | 273,930 | 0.491% |
| Screening birth cohort: before 1945 | | |
| Province | Population size | Minimum Prevalence (Threshold) |
| AB | 315,558 | Not cost-effective |
| BC | 538,475 | Not cost-effective |
| MB | 129,202 | Not cost-effective |
| NB | 93,295 | Not cost-effective |
| NL | 59,858 | Not cost-effective |
| NS | 115,687 | Not cost-effective |
| ON | 1,472,090 | Not cost-effective |
| PE | 17,167 | Not cost-effective |
| QC | 958,718 | Not cost-effective |
| SK | 114,610 | Not cost-effective |

**References**

1. Wong WWL, Erman A, Feld JJ, Krahn M. Model-based projection of health and economic effects of screening for hepatitis C in Canada. CMAJ Open. 2017;5(3):E662-E72. doi: 10.9778/cmajo.20170048.

2. Miller CL, Kerr T, Strathdee SA, Li K, Wood E. Factors associated with premature mortality among young injection drug users in Vancouver. Harm Reduct J. 2007;4:1-. doi: 10.1186/1477-7517-4-1.

3. Vondeling GT, Cao Q, Postma MJ, Rozenbaum MH. The Impact of Patent Expiry on Drug Prices: A Systematic Literature Review. Appl Health Econ Health Policy. 2018;16(5):653-60. doi: 10.1007/s40258-018-0406-6.

4. Major D, Bryant H, Delaney M, Fekete S, Gentile L, Harrison M, et al. Colorectal cancer screening in Canada: results from the first round of screening for five provincial programs. Curr Oncol. 2013;20(5):252-7. doi: 10.3747/co.20.1646.
